# Cost-effectiveness of the screen-and-treat strategies using HPV test linked to thermal ablation for cervical cancer prevention in China: a modeling study

**DOI:** 10.1186/s12916-023-02840-8

**Published:** 2023-04-17

**Authors:** Xue-Lian Zhao, Shuang Zhao, Chang-Fa Xia, Shang-Ying Hu, Xian-Zhi Duan, Zhi-Hua Liu, Yue-Yun Wang, Ting-Ting You, Meng Gao, You-Lin Qiao, Partha Basu, Fang-Hui Zhao

**Affiliations:** 1 Department of Epidemiology, National Cancer Center/National Clinical Research Center for Cancer/Cancer Hospital, Chinese Academy of Medical Sciences and Peking Union Medical College, Beijing, China; 2 Department of Clinical Trial Research Center, Beijing Hospital, National Center of Gerontology, Institute of Geriatric Medicine, Chinese Academy of Medical Sciences, Beijing, China; 3 Office of Cancer Screening, National Cancer Center/National Clinical Research Center for Cancer/Cancer Hospital, Chinese Academy of Medical Sciences and Peking Union Medical College, Beijing, China; 4 Department of Obstetrics and Gynecology, Beijing Tongren Hospital, Beijing, China; 5 Affiliated Shenzhen Maternity and Child Healthcare Hospital, Southern Medical University, Shenzhen, China; 6 School of Population Medicine and Public Health, Chinese Academy of Medical Sciences and Peking Union Medical College, Beijing, China; 7 Early Detection, Prevention & Infections Branch, International Agency for Research on Cancer, Lyon, France

**Keywords:** Cervical cancer, Screen and treat, HPV test, Thermal ablation, Cost-effectiveness

## Abstract

**Background:**

Self-sampling HPV test and thermal ablation are effective tools to increase screening coverage and treatment compliance for accelerating cervical cancer elimination. We assessed the cost-effectiveness of their combined strategies to inform accessible, affordable, and acceptable cervical cancer prevention strategies.

**Methods:**

We developed a hybrid model to evaluate costs, health outcomes, and incremental cost-effectiveness ratios (ICER) of six screen-and-treat strategies combining HPV testing (self-sampling or physician-sampling), triage modalities (HPV genotyping, colposcopy or none) and thermal ablation, from a societal perspective. A designated initial cohort of 100,000 females born in 2015 was considered. Strategies with an ICER less than the Chinese gross domestic product (GDP) per capita ($10,350) were considered highly cost-effective.

**Results:**

Compared with current strategies in China (physician-HPV with genotype or cytology triage), all screen-and-treat strategies are cost-effective and self-HPV without triage is optimal with the most incremental quality-adjusted life-years (QALYs) gained (220 to 440) in rural and urban China. Each screen-and-treat strategy based on self-collected samples is cost-saving compared with current strategies (−$818,430 to −$3540) whereas more costs are incurred using physician-collected samples compared with current physician-HPV with genotype triage (+$20,840 to +$182,840). For screen-and-treat strategies without triage, more costs (+$9404 to +$380,217) would be invested in the screening and treatment of precancerous lesions rather than the cancer treatment compared with the current screening strategies. Notably, however, more than 81.6% of HPV-positive women would be overtreated. If triaged with HPV 7 types or HPV16/18 genotypes, 79.1% or 67.2% (respectively) of HPV-positive women would be overtreated with fewer cancer cases avoided (19 cases or 69 cases).

**Conclusions:**

Screen-and-treat strategy using self-sampling HPV test linked to thermal ablation could be the most cost-effective for cervical cancer prevention in China. Additional triage with quality-assured performance could reduce overtreatment and remains highly cost-effective compared with current strategies.

**Supplementary Information:**

The online version contains supplementary material available at 10.1186/s12916-023-02840-8.

## Background

The global action towards the elimination of cervical cancer can be considered as a historic milestone in our fight against cancer. In 2020, World Health Organization (WHO) launched the global strategy to accelerate cervical cancer elimination with ambitious intermediate targets on screening and treatment by 2030, i.e., achieving 70% screening coverage with a high-performance test and 90% of women with a positive screening test or a cervical lesion managed appropriately [1]. Several modeling studies have demonstrated the indispensable role of scaling-up screening and treatment in achieving the target of cervical cancer elimination, since vaccination against human papillomaviruses (HPV) alone will not be adequate to achieve the desired milestones [2–4].

A huge gap exists between the current status of cervical screening and 2030 targets in the developing countries. The wide variation in the prevalence of cervical cancer screening was reported from 57 countries worldwide and majority (67.3%) of low- and middle-income countries (LMICs) are far away from the target of 70% cervical screening coverage [5]. According to the WHO NCD Country Capacity Survey, only 50% of the middle-income countries have the ability to provide comprehensive cancer diagnosis and treatment services; the proportion being even less in low-income countries [6]. Identifying strategies, which are effective as well as affordable and acceptable to be rolled up nationwide, should be key areas of focus to increase the accessibility of the cervical screening and treatment to reach the 2030 targets.

HPV testing on self-collected samples (self-sampling), thermal ablation to treat cervical intraepithelial neoplasia (CIN), and screen-and-treat strategies using these technologies have the potential to increase screening coverage and treatment compliance of cervical cancer screening programs [7, 8]. Our previous studies have reported the effectiveness and feasibility in applying them in the population-based cervical cancer screening program in China [9, 10]. However, limited analyses have demonstrated its cost-effectiveness when introduced into the current health system for cervical cancer prevention. In the present research, we will model and assess the cost-effectiveness of a series of screen-and-treat algorithms using HPV tests linked to thermal ablation in comparison with currently used strategies in China, providing scientific evidence for its introduction in the global screening strategy for cervical cancer elimination.

## Methods

### Overview

We conducted a model-based economic evaluation to assess the cost-effectiveness of six screen-and-treat strategies combining primary HPV testing (self-sampling or physician-sampling), triage modalities (HPV genotyping, colposcopy or no triage) and thermal ablation treatment in comparison with currently used screening strategies in China, from a societal perspective. For these strategies, we simulated a single cohort of 100,000 females born in 2015 from birth to death (life expectancy 85 years), as the specified screen and treatment pathways, to obtain the estimates of costs and health outcomes. Screening was included as the intervention for cervical cancer prevention and the vaccination was not considered in the model due to the negligible HPV vaccination coverage in China currently [11].

### Model

We updated our previously developed hybrid model, and its structure is shown in the Additional file: Fig. S1 [4, 12]. Briefly, the model consisted of a dynamic model and a natural history model. We used the dynamic model to simulate the HPV transmission between males and females, and another model to simulate the natural history of cervical cancer and to obtain the number of cervical precursors or cancer cases and deaths associated with HPV infections. The two-stage hybrid model was concatenated by the force of infection—i.e., the age-specific HPV incidence from the dynamic model served as inputs to the natural history model. Each individual was entered into the model at their birth age and randomly allocated to a new state, based on the transition probabilities (Additional file: Table S1) [13–21]. Individuals were transitioned among states representing HPV infection, CIN grade 1, CIN2, CIN3, and cervical cancer. Cervical cancer screening fitted in the natural history model where individuals were randomly assigned to screening. In the absence of screening, women with CINs or cervical cancer would be diagnosed when the related symptoms develop. Diagnosed individuals would receive treatment specific to the stage of the disease. We assumed that women with cervical cancer are subjected to the stage-specific mortality rates in addition to all-cause mortality rates. The model was built with a cycle length of one year.

The model was calibrated using epidemiological data of HPV prevalence, cervical cancer incidence and mortality in 2015, HPV genotype distributions in women with normal cervical cytology, low-grade squamous intraepithelial lesion, high-grade squamous intraepithelial lesion, and invasive cervical cancer [22–26]. The model was validated using the reported prevalent risk and 5-year cumulative risk of CIN2 or worse (CIN2+) after one positive HPV test, and 10-year cumulative detection rate of CIN2+ in Chinese women (Additional file: Fig. S2) [27]. The model tracked disease progression and regression, clinical events, lifetime economic outcomes, and health outcomes. Further details of this model were described in the Additional file and article published earlier [4, 12].

### Screening scenarios

The assumed standard-of-care strategy included screening women with HPV testing using samples collected by physicians, followed by triaging with HPV16/18 genotyping or cytology (at a threshold of atypical squamous cell of undetermined significance or ASCUS), and treatment for the histologically confirmed CIN2+ lesions with loop electrosurgical excision procedure (LEEP). We considered six screen-and-treat strategies as alternative scenarios. These included combinations of HPV testing (either self-sampled or physician-sampled), triage modalities (HPV genotyping and colposcopy in various combinations), and thermal ablation of screen-positive or triage-positive women (Table 1 and please see Additional file for more details ).

**Table 1:** Scenarios for all screen-and-treat and currently used strategies in China

**Scenarios**	**Strategies**	**Screening test**	**Triage test**	**Treatment method**
**1**	Self-HPV without triage	Self-HPV test	/	Thermal ablation^a^
**2**	Self-HPV16/18 triage	Self-HPV test	HPV16/18 positive → thermal ablation; Other hrHPV positive → triage by colposcopy	Thermal ablation^a^
**3**	Self-HPV7 types triage^b^	Self-HPV test	HPV16/18/31/33/45/52/58 positive → thermal ablation; other hrHPV positive → triage by colposcopy	Thermal ablation^a^
**4**	Physician-HPV without triage	Physician-HPV test	/	Thermal ablation^a^
**5**	Physician-HPV16/18 triage	Physician-HPV test	HPV16/18 positive → thermal ablation; other hrHPV positive → triage by colposcopy	Thermal ablation^a^
**6**	Physician-HPV7 types triage^b^	Physician-HPV test	HPV16/18/31/33/45/52/58 positive → thermal ablation; other hrHPV positive → triage for colposcopy	Thermal ablation^a^
**7 (reference)**	Physician-HPV with genotype triage	Physician-HPV test, cytology	HPV16/18 positive→ referred to colposcopy; other hrHPV positive → triage by cytology, women with ASCUS+ referred to colposcopy	LEEP
**8 (reference)**	Physician-HPV with cytology triage	Physician-HPV test, cytology	hrHPV positive → triage by cytology, women with ASCUS+ referred to colposcopy	LEEP

^a^Eligibility for thermal ablation is an assessment for women with a positive screening test under colposcopy. Women receive thermal ablation if there is no suspicion of invasive or glandular disease, and meet the following criteria: the transformation zone (TZ) is fully visible, the whole lesion is visible and it does not extend into the endocervix, or the lesion is type 1 TZ, or the lesion is type 2 TZ where the probe tip will achieve complete ablation of the SCI epithelium, i.e., where it can reach the upper limit of the TZ. Sometimes the SCJ can be seen high in the canal but a probe tip would not reach it

^b^Seven types of hrHPV referred to HPV16, 18, 31, 33, 45, 52, 58

*hrHPV*, high risk human papillomavirus; *ASCUS+*, atypical squamous cells of undetermined significance or above; *LEEP*, loop electrosurgical excision procedure

The target population for cervical cancer screening was women aged 30−65 years in urban and rural China, with a 5-year screening interval. The screening, triage, and treatment procedures were shown in the supplementary file (Additional file: Fig. S3a−h, Fig. S4). We assumed that the anticipated screening coverage would reach 70% with physician-collected samples, and 89.6% (95% CI 63.0 to 100.0%) with self-collected samples according to the ratio of screening uptake reported in a meta-analysis (self-sampling participation versus physician-sampling, RR=1.28, 95% CI 0.90 to 1.82) [28]. Triage, diagnostic, and treatment compliance were also included in the model, which was assumed to reduce by 15% for each additional visit [29].

### Inputs and assumptions

Base-case estimates and ranges for all parameters were listed in Table 2 and Additional file [4, 9, 10, 12–21, 23–26, 28–33]. Natural history parameters of cervical cancer were extracted from the national statistical databases and literature reviews (Additional file: Table S1) [13–21]. The proportion of residents with several sexual partners were extracted from a national representative longitudinal study [34]. The prevalence of high-risk HPV infections was extracted from a pooled study in China [35]. The sensitivity and specificity of different strategies were calculated based on large-scale population-based studies in China [9, 31, 32]. The efficacy of thermal ablation and LEEP treatment was collected from open-source publications [10, 33].

**Table 2:** Parameters used in the model analysis

Parameter	CIN1 Base case (95%CI)	CIN2 Base case (95%CI)	CIN3 Base case (95%CI)	Distribution	Source
**Efficacy of screening strategies**					
Self-HPV without triage					[9]
Sensitivity	0.70(0.61–0.77)	0.84(0.76–0.89)	0.85(0.75–0.91)	Beta	
Specificity	0.83(0.82–0.84)	0.82(0.81–0.83)	0.81(0.80–0.82)	Beta	
Self-HPV16/18 triage					
Sensitivity	0.41(0.32–0.52)	0.67(0.57–0.75)	0.72(0.61–0.82)	Beta	
Specificity	0.97(0.96–0.97)	0^.^96(0^.^96–0^.^97)	0.95(0.95–0.96)	Beta	
Self-HPV7 types triage					
Sensitivity	0.60(0.51–0.68)	0.80(0.72–0.87)	0.81(0.70–0.88)	Beta	
Specificity	0.89(0.88–0.90)	0.88(0.88–0.89)	0.88(0.87–0.88)	Beta	
Physician-HPV without triage					[31, 32]
Sensitivity	0.76(0.53–0.90)	0.84(0.62–0.94)	0.90(0.74–0.97)	Beta	
Specificity	0.82(0.80–0.83)	0.81(0.80–0.83)	0.81(0.79–0.82)	Beta	
Physician-HPV16/18 triage					
Sensitivity	0.59(0.36–0.78)	0.53(0.32–0.73)	0.76(0.56–0.88)	Beta	
Specificity	0.96(0.95–0.97)	0.95(0.94–0.96)	0.95(0.94–0.96)	Beta	
Physician-HPV7 types triage					
Sensitivity	0.65(0.41–0.83)	0.84(0.62–0.94)	0.86(0.69–0.95)	Beta	
Specificity	0.88(0.86–0.89)	0.87(0.86–0.89)	0.86(0.85–0.88)	Beta	
Physician-HPV with genotype triage					[31, 32]
Sensitivity	0.81(0.57–0.93)	0.98(0.89–1.00)	0.97(0.83–0.99)	Beta	
Specificity	0.59(0.54–0.64)	0.57(0.52–0.62)	0.54(0.49–0.59)	Beta	
Physician-HPV with cytology triage					
Sensitivity	0.50(0.28–0.72)	0.84(0.62–0.94)	0.82(0.64–0.92)	Beta	
Specificity	0.66(0.61–0.71)	0.66(0.61–0.70)	0.63(0.58–0.68)	Beta	
**Participation variables**	**Base case (Range)**				
Participation of physician-sampling	0.70(0.25–1.00)			Beta	Assumed
RR (self-sampling participation vs physician-sampling)	1.28(0.90–1.82)			Ln (RR) is normal	[28]
Loss to follow-up (per visit)	0.15(0.00–0.50)			Beta	[29]
**Treatment efficacy**					
Thermal ablation for HPV positive	0.804(0.734–0.859)			Beta	[10]
Thermal ablation for CIN 1	0.903(0.805–0.955)			Beta	[10]
Thermal ablation for CIN 2/3	0.762(0.615–0.865)			Beta	[10]
LEEP for HPV positive	/				
LEEP for CIN 2/3	0.947(0.931–0.963)			Beta	[33]
**Eligibility for thermal ablation**					
HPV positive, without CIN	0.456(0.342–0.570)			Beta	[9, 30]
CIN 1	0.510(0.383–0.638)			Beta	
CIN 2	0.534(0.400–0.667)			Beta	
CIN 3	0.449(0.337–0.561)			Beta	
**Precancer management for current strategies**					
***Urban area***					
Follow up and management for CIN 1	0.905(0.815–0.996)			Beta	[4, 12]
Treatment of CIN2/3	0.953(0.858–1.000)			Beta	[4, 12]
***Rural area***					
Follow up and management for CIN 1	0.841(0.757–0.925)			Beta	[4, 12]
Treatment of CIN2/3	0.895(0.805–0.984)			Beta	[4, 12]
**Treatment costs (2020 US$)**					
TA	11.43(± 25%)			Gamma	Micro-costing approach
LEEP	155.63(± 25%)			Gamma	[36]
**Costs of cervical cancer treatment (2020 US$)**					
Urban area					
CC FIGOI-IIa treatment	7974.19(± 25%)			Gamma	[4, 36–38]
CC FIGO IIb-IV treatment	14,051.52(± 25%)			Gamma	[4, 36–38]
Rural area					
CC FIGOI-IIa treatment	5329.05(± 25%)			Gamma	[4, 36–38]
CC FIGO IIb-IV treatment	8819.70(± 25%)			Gamma	[4, 36–38]
**Follow up cost after treatment (2020 US$)**					
**Urban area**					
TA for self-sampling strategy (HPV positive)	33.73(± 25%)			Gamma	[10], Micro-costing approach
TA for self-sampling strategy (CIN1 +)	49.27(± 25%)			Gamma	
TA for physician-sampling strategy (HPV positive)	47.67(± 25%)			Gamma	
TA for physician-sampling strategy (CIN1 +)	64.80(± 25%)			Gamma	
LEEP (CIN2 or CIN3)	106.92(± 25%)			Gamma	
**Rural area**					
TA for self-sampling strategy (HPV positive)	29.90 (± 25%)			Gamma	[10], Micro-costing approach
TA for self-sampling strategy (CIN1 +)	45.23 (± 25%)			Gamma	
TA for physician-sampling strategy (HPV positive)	39.17(± 25%)			Gamma	
TA for physician-sampling strategy (CIN1 +)	55.57(± 25%)			Gamma	
LEEP (CIN2 or CIN3)	97.73 (± 25%)			Gamma	
**Utility**					
Utility before thermal ablation	0.986(0.978–0.994)			Normal	[39, 40]
Utility after thermal ablation					
≤ CIN1	0.989(0.983–0.996)			Normal	
CIN2 +	0.965(0.930–0.999)			Normal	
Utility before LEEP	0.984(0.977–0.992)			Normal	
Utility after LEEP	0.956(0.938–0.974)			Normal	

More details about the related parameters have been represented in the Additional file 1, pp 12-24

*HPV* Human papillomavirus, *TA* Thermal ablation, *CIN* Cervical intraepithelial neoplasia, *RR* Relative risk (risk ratio) comparing self-collection with provider collection of samples for cervical cancer screening, *LEEP* Loop electrosurgical excision procedure, *FIGO* International Federation of Gynecology and Obstetric

Costs of cervical cancer screening scenario included the costs of screening, treatment, and administration (Table 2 and Additional file: Table S2a−h, Table S3) [4, 10, 22, 35–38, 41–44]. Cervical screening costs were calculated according to the government-provided cost calculation table and our population-based pooled data [36, 41–44]. The cost of thermal ablation treatment was calculated using micro-costing approach considering the cost and life span of the equipment as well as the personnel cost for each treatment, since the technology has not been introduced to the routine clinical practice in China (see Additional file for more details). The cost of LEEP treatment was derived from the average charges in the secondary facilities in China [36]. All components of direct medical cost, direct non-medical costs, and indirect costs for women receiving screening and treatment were considered in the model [36–38]. Costs were converted into US dollars using exchange rates for early 2020 (i.e., 1.00 US dollar = 7.00 Chinese yuan).

Utility scores stratified by the thermal ablation or LEEP treatment were obtained from a multicenter population-based cervical cancer screening program using the quality-of-life (QOL) assessments questionnaire, which was conducted in both rural and urban settings in 2021 (Table 2 and Additional file: Table S4) [39, 40].

### Outcomes

Using the calibrated model, we estimated the lifetime costs and health benefits for each strategy in rural and urban China. The cost of implementing each strategy was estimated from a societal perspective. The health outcomes of each strategy were evaluated in quality-adjusted life-years (QALYs), taking into account health state utility weights. Both costs and health outcomes were discounted at an annual rate of 3% with a range of 0% to 5% tested in a sensitivity analysis [45]. We calculated the incremental cost-effectiveness ratio (ICER), defined as the incremental cost per QALY gained for each strategy compared with the currently used screening strategy, to identify the cost-effective strategy. Here, we applied the Chinese gross domestic product (GDP) per capita ($10,350 in 2020) as the cost-effectiveness frontier (highly cost-effective, cost-effective, or not cost-effective with an ICER <1, 1−3, or >3-times the per-capita GDP) [46]. We also evaluated the harm-and-benefit tradeoff of each screening strategy (presented by the numbers of over-treatment versus the numbers of CIN1+, CIN2+, or CIN3+ detected). Overtreatment rate was defined as the proportion of women treated by thermal ablation who did not have any CIN on histopathology.

### Sensitivity analysis

We did one-way deterministic sensitivity analyses by varying each input value in the model over a plausible range to examine the impact of uncertainty in individual input parameters on the results. We conducted probabilistic sensitivity analysis by performing 10,000 Monte Carlo simulations to sample parameter values from their distributions and estimate outcomes. Cost-effectiveness acceptability curves were conducted to compare screen-and-treat strategies with currently used strategies across a wide range of willing-to-pay (WTP) thresholds.

## Results

**Fig. 1:**
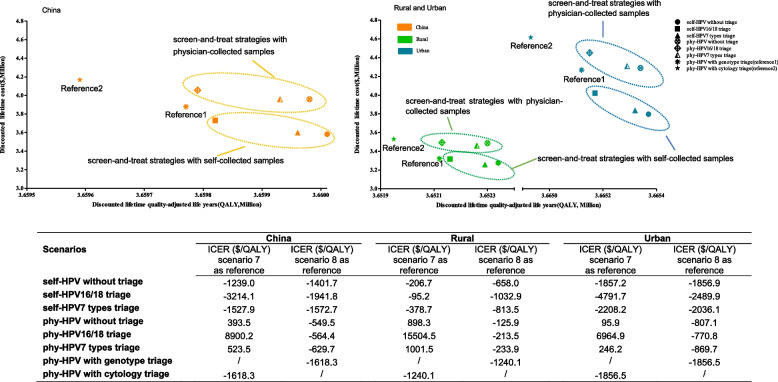
Cost-effectiveness analysis for all screen-and-treat strategies versus the currently used strategies. HPV, human papillomavirus; QALY, quality-adjusted life-years; phy, physician; ICER, incremental cost-effectiveness ratio

First for all of China, in comparison to the currently used strategies (physician-collected HPV test with genotype and cytology triage), all screen-and-treat strategies are highly cost-effective with the discounted ICERs ranging from −$3214.1 to 8900.2 per QALY gained (Fig. 1 and Additional file: Table S5a−b ). Compared with the physician-collected HPV test with cytology triage strategy, all screen-and-treat strategies are cost-saving (−$586,290 to −$112,540) with more QALYs yielded (200 to 420), and self-collected HPV test without triage is regarded as the optimal strategy with the most incremental QALYs gained (420 QALYs; ICER= −$1401.7 per QALY), followed by the physician-collected HPV test without triage, self-HPV 7 types triage, physician-HPV 7 types triage, self-HPV16/18 triage, and physician-HPV16/18 triage. The situation is basically the same when regarding physician-HPV with genotype triage as the reference, more QALYs gained (20 to 240) at lower costs (−$295,890 to −$146,630) with self-collected samples whereas at higher costs (+$80,030 to +$177,860) with physician-collected samples.

For urban and rural areas, likewise, each screen-and-treat strategy is highly cost-effective in urban areas (ICERs= −$4791.7 to 6964.9 per QALY) with more QALYs yielded at a slightly higher cost than that in rural areas. Self-HPV without triage is the optimal strategy for both urban and rural areas with the most incremental QALYs gained (220 to 440). Physician-HPV16/18 triage leads to the most incremental costs compared with the current strategies but still cost-effective in urban and rural areas (ICERs= $6964.9 and 15,504.5 per QALY).

**Fig. 2:**
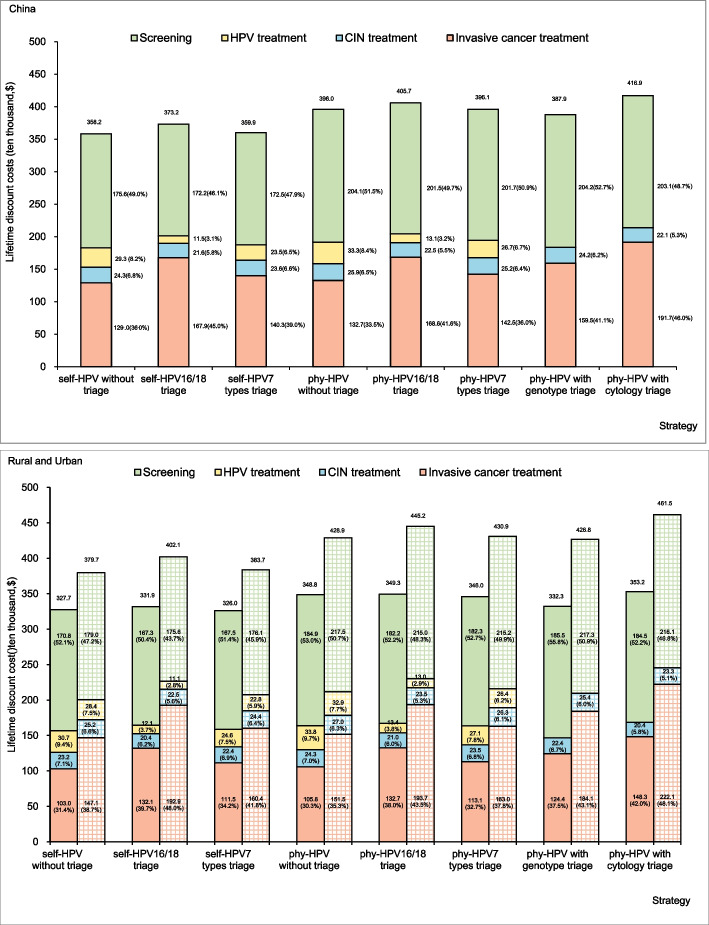
Discounted costs of each cervical screening strategy over the lifetime, broken down by component. Note: Solid bars represent rural areas, and shaded bars represent urban areas. HPV, human papillomavirus; CIN, cervical intraepithelial neoplasia; phy, physician

The discounted costs of each cervical screening strategy over the lifetime are shown in Fig. 2, which is broken down by the components of screening costs, treatment costs for HPV infection, CINs, and cervical cancer. The lifetime costs range from $3.6 to 3.7 million for screen-and-treat strategies with self-collected samples, $4.0–4.1 million for screen-and-treat strategies with physician-collected samples, $3.9 million for current physician-HPV with genotype triage, to $4.2 million for current physician-HPV with cytology triage. It is estimated that 41.1% and 46.0% of costs are attributed to cervical cancer treatment with current physician-HPV with genotype triage or cytology triage strategies, which reduce by 5.1–7.6% and 10.0–12.5% for screen-and-treat strategies without triage, 2.1–5.1% and 7.0–10.0% for screen-and-treat strategies with 7 types triage, accompanying with the increasing HPV and CINs treatment costs. Yet, the cervical cancer treatment costs remain high for screen-and-treat strategies with HPV16/18 triage. Similar patterns are observed for urban and rural areas.

**Fig. 3:**
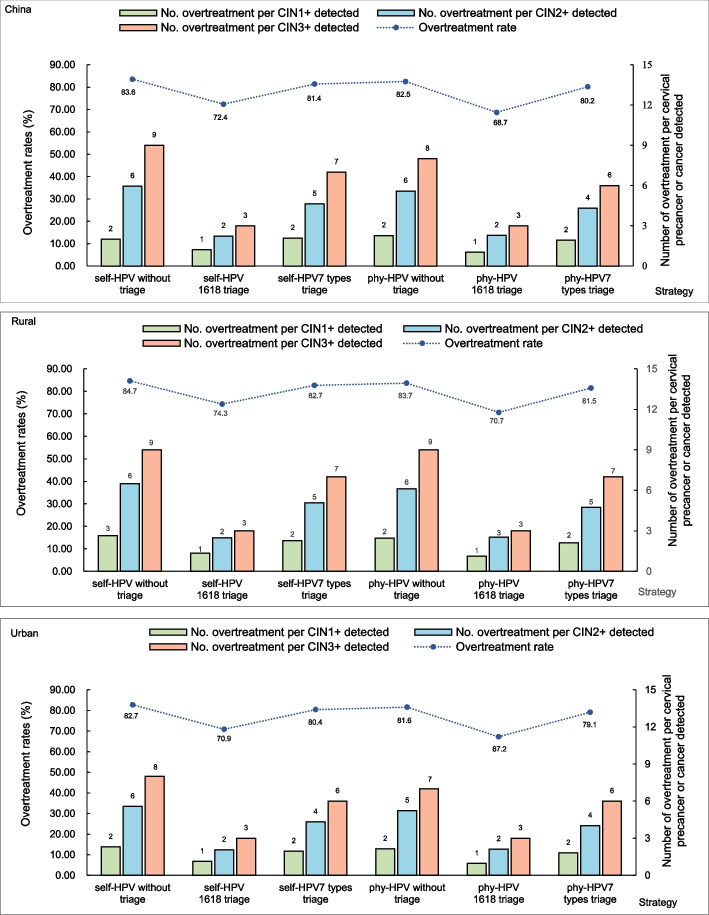
Overtreatment rates and harm-and-benefit tradeoff for each screen-and-treat strategy. HPV, human papillomavirus; CIN, cervical intraepithelial neoplasia; phy, physician

We assess overtreatment rates as well as the number of women overtreated per CINs or worse lesion detected in each screen-and-treat strategy to identify the benefits and harms tradeoff (Fig. 3). More than 6379 (67.2%) women would be overtreated using the screen-and-treat strategies in rural and urban China, with the highest overtreatment rates of more than 81.6% in strategies without triage (self- or physician-sampled; more than 16,622 women overtreated). If triaged with respect to HPV 7 types or HPV16/18 genotypes, 79.1% (13,093 women) or 67.2% (6,379 women) of HPV-positive women would be overtreated with fewer cancer cases avoided (19 cases or 69 cases). Accordingly, the largest number of women would be overtreated per CIN2+ or CIN3+ detected using screen-and-treat strategies without triage (5 to 9 overtreated), followed by strategies with HPV 7 types triage (4 to 7 overtreated) and HPV16/18 triage (2 to 3 overtreated). A similar number of overtreatment per CIN1+ detected could be observed between strategies without triage and with HPV 7 types triage (2 to 3 overtreated), which is higher than strategies with HPV16/18 triage (1 overtreated).

**Fig. 4:**
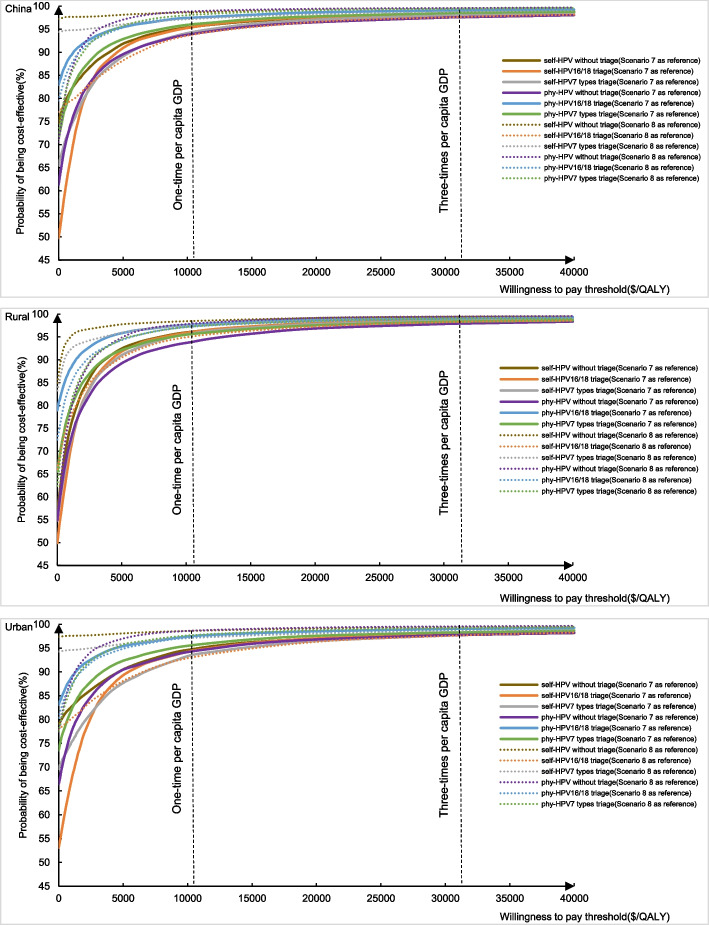
Cost-effectiveness acceptability curves for all screen-and-treat strategies. HPV, human papillomavirus; GDP, gross domestic product; phy, physician

Figure 4 shows the cost-effectiveness acceptability curves for all screen-and-treat strategies at a range of WTP thresholds between 0 and three times per-capita GDP. At a WTP threshold of three times per-capita GDP, at least a 97.6% probability of being cost-effective for all the screen-and-treat strategies, which remain at least 93.8% when the WTP reduce to one time per-capita GDP.

**Fig. 5:**
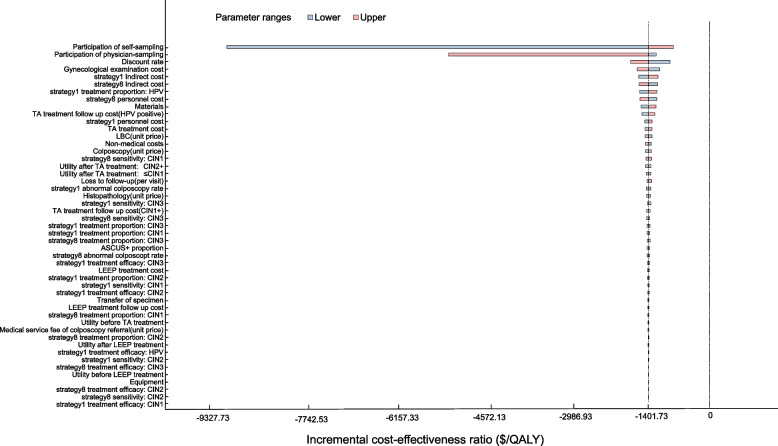
Tornado diagram analysis for optimal strategy (self-HPV without triage) versus traditional strategy (physician-HPV with cytology) in China. QALY, quality-adjusted life-years; TA, thermal ablation; CIN, cervical intraepithelial neoplasia; LEEP, loop electrosurgical excision procedure; phy, physician

The findings of our analysis are most sensitive to the participation rates of a cervical screening program with self-collected samples and physician-collected samples, as well as the discount rate. Input to which the results are most sensitive was participation rates with self-collected samples in both urban and rural areas, which potentially increase the ICER to -$1357.0 per QALY in urban areas and −$247.4 per QALY in rural areas (Fig. 5 and Additional file: Fig. S5a−b ).

## Discussion

Our research comprehensively analyzed, the cost-effectiveness of the screen-and-treat strategies combining self-sampling HPV test and thermal ablation to identify the effective, affordable as well as acceptable strategy for cervical screening and treatment. We found that compared with the currently used strategies, all screen-and-treat strategies were cost-effective and self-HPV without triage, i.e., primary HPV testing with self-collected samples followed by the immediate thermal ablation treatment for the HPV-positive women, was the optimal strategy with the most QALYs gained and the most costs saved in both rural and urban China. Further, the costs for cervical cancer treatment among the whole population could be significantly reduced by the screen-and-treat strategies without triage or with HPV 16/18/31/33/45/52/58 triage, and the resources may be better utilized towards implementing screening and precancer treatment.

Health economic evaluation is essential to select the most appropriate strategies among the “cafeteria” of different choices currently available from a decision-making perspective. However, such evaluation has not been modeled on utilizing the screen-and-treat strategy with self-sampling HPV test linked to thermal ablation into the population-based program. In 2021, the WHO updated guidelines recommended HPV test-based screen-and-treat strategies for screening and treatment of cervical precancer lesions in the general population. Meanwhile, HPV testing by self-sampling and thermal ablation treatment were recommended as possible approaches to further scale up the services [47]. Our analysis took full account of costs incurred in the practical implementation of the self-sampling HPV test-based screen-and-treat program with thermal ablation, as well the effectiveness derived from the local population-based studies [9, 10]. Compared with the currently used strategies in most of the countries, including China, we found that all the HPV test-based screen-and-treat strategies with self-collected samples and thermal ablation showed obvious advantages in not only for cost-saving but more QALYs gained simultaneously. Further considering its advantages in high screening and treatment performance, portability of equipment, conserving health resources and manpower, achieving adequate population coverage as well as providing timely management of screen-positive results for the cervical screening program, such strategy has the great potential to help reach the 2030 targets with maximizing the accessibility of the health care services for cervical cancer prevention [8, 48, 49].

Notably, in our study, we used GDP per capita as a cost-effectiveness threshold, which was commonly used worldwide and adopted in the WHO guidelines on Choosing Interventions that are Cost Effective (WHO-CHOICE) [46]. Currently, there is a move to use opportunity cost-based thresholds to assess cost-effectiveness of interventions, which was regarded as more in accord with countries’ realities than the GDP per capita in low- and middle-income countries [50]. If using half of GDP per capita, a conservative opportunity cost-based threshold, the screen-and-treat strategy of physician sampling HPV16/18 triage strategy would not be cost-effective. That might remind us that the adoption of this intervention should be paid more attention to investigate whether it is in practice locally affordable.

In the context of the fight against cancer, prevention is always better than cure, and never has the opportunity been greater, especially for cervical cancer prevention [51]. The biggest challenge that we face is ensuring that opportunities for health gain are delivered to the largest number who could benefit. A modeling study has reported that, under the Chinese current screening strategy with low coverage, the cervical cancer incidence is projected to increase with more expenditures mainly spent on the treatment of invasive cervical cancer [4, 12]. After more investments in the HPV vaccine and scaling-up of cervical screening, more than 7.5 million cervical cancer cases would be further averted before 2100, and the total cost for cervical cancer prevention would drop sharply due to the cost reduction in cervical cancer treatment [4]. Likewise, our research reported that the costs for the cervical cancer treatment can be reduced by 2.1–12.5% in utilizing the screen-and-treat strategies without triage or triaging with the most common high-risk HPV genotypes in the population, permitting more resources to be allocated to screening and CINs treatment. The findings indicated that expanding screening based on self-collected samples while strengthening its link to the immediate thermal ablation treatment could contribute to the cost-saving as well as the cancer control strategy moving from cancer treatment towards cancer prevention.

A certain amount of overtreatment is inevitable in the screen-and-treat approach, which constitutes the major concern impeding its implementation in some countries, such as China [52]. Encouragingly, thermal ablation, as a promising method used in the screen-and-treat strategies, has been demonstrated similar treatment success to cryotherapy as well as loop excision with few adverse effects and complications [8, 10]. Additionally, our 3-year prospective study has indicated that thermal ablation could clear HPV infection by 73% among HPV-positive women [53]. Thus, treatment of HPV-positive women may not be considered as ‘overtreatment’ but a simple and safe means to prevent future risk of cervical cancer. Our analysis took into account the impacts of the overtreatments on QOL, and the final results demonstrated that the benefits outweighed the harms arising from the overtreatment in utilizing the thermal ablation in the screen-and-treat strategies. But up until now, the long-term effect (more than 3 years) of the overtreatment on women’s health with the thermal ablation treatment remains unclear. In this regard, triage to reduce overtreatment by identifying the high-risk population in developing cervical cancer is generally regarded as the most effective measure. Our study reported that five to nine women would be overtreated per CIN2+ or CIN3+ detected using screen-and-treat strategies, which could be further reduced by HPV genotyping triage with a few CIN2+ being missed. In practice, local health-related decision-makers may determine the acceptable overtreatment threshold and select the triage strategy most affordable and feasible in the local setting. Further, prospective follow-up is required to better understand the benefits and harms tradeoff among all the screen-and-treat strategies.

In China, the traditional multi-visit strategy with screening, colposcopy, and biopsy for diagnosis, and treatment for pathological confirmed CIN2+ has been widely implemented in the national cervical cancer screening program [54]. But the comprehensive requirements for resources and capacity building precludes its roll-up in economically underdeveloped areas [55]. Under such circumstances, screen-and-treat strategies, as a supplement of the current screening practice, should be considered to apply in the low-recourse areas and delivered by the local primary care system. The Chinese government has released favorable policies in promoting investment and enhancing the construction of the primary health care (PHC) system since 2009. As of 2017, the subsidies as a proportion of total PHC income increased from 12.3% to 32.5% [56]. The related actions signal the government’s attention to universal health as well as provide unprecedented opportunities in delivering screen-and-treat services in the primary facilities, further reducing the national disparities and inequity in cervical cancer prevention. Therefore, for countries with established national cervical cancer screening programs, such as China, the simple and labor-saving screen-and-treat strategies would further scale up the current screening services to achieve the 70% and 90% targets. Meanwhile, for the other 35% of the countries which are lack of national cervical cancer screening programs worldwide [6], such strategies contribute to accelerate the initiation of national cervical cancer screening and treatment services.

Regarding to cost-effectiveness studies of screen-and-treat strategies, the most widely evaluated strategies were based on HPV testing with physician-collected samples followed by cryotherapy. In a comparative study in El Salvador, HPV-based screening followed by cryotherapy treatment is considered cost-effective [57]. Another modeling study indicated that the one-visit screen-and-treat strategy (HPV test followed by same-day cryotherapy) facilitated by point-of-care technology generated greater benefits than the two-visit approach (requiring a return visit for treatment), especially in areas with high loss to follow-up [58]. Likewise, a cost-effectiveness study conducted in Kenya also showed that HPV screening may become less expensive than a visual inspection with acetic acid (VIA) if it could be reduced to a single visit. Preventative cryotherapy was the least expensive strategy and led to the highest projected life expectancy [59]. However, no study ever evaluated the cost-effectiveness of screen-and-treat strategies based on self-sampling HPV tests combined with thermal ablation up until now. Some triage methods, such as VIA, have been assessed in health economics evaluation studies. In a cost-effectiveness analysis, HPV with VIA triage cost more but was less effective than HPV alone in settings with high cervical cancer burden, due to the VIA triage missing some precancers that were destined to progress [60]. Self-sampling HPV test alone followed by immediate thermal ablation may achieve greater health benefits with relatively lower costs. However, limited information is available about its evaluations on health economics.

To the best of our knowledge, our study is the first attempt to evaluate the cost-effectiveness of the screen-and-treat strategies combining self-sampling HPV test and thermal ablation in China, which makes strong evidence for supporting its use in cervical cancer prevention. Further, our analysis has considered costs, effects as well as overtreatments over the full spectrum of the screening program, which provides a comprehensive and objective information to inform the policy marking on cancer prevention. Meanwhile, our research has several limitations. First, the parameters included in the model analysis were mostly derived from the local population-based research in China, restricting the generalization of the relevant findings in other countries to some extent. Second, long-term health consequences such as fertility and obstetrical outcomes resulting from the treatment were not considered in the analysis due to the absence of related evidence on thermal ablation globally. Third, we did not consider the costs of training for thermal ablation treatment, which is relatively minimal due to the procedure is simple to perform that can be done by trained midwives, nurses, and other medical personnel without anesthesia [10]. Last, HPV vaccination was not considered in the model analysis. In our study, we aim to evaluate the cost-effectiveness of six screen-and-treat strategies based on the current cervical cancer prevention setting in China. Currently, China has not introduced the HPV vaccination into its national program, and the vaccination coverage in China is very low [11]. However, we do admit that China may initiate its national HPV vaccination program in the future. Not considering the HPV vaccination may lead to an overestimate of the burden that can be prevented by screening and treatment over the long term.

## Conclusions

Our research has demonstrated that the screen-and-treat strategy linking self-sampling HPV test with the immediate thermal ablation treatment for the screen-positive women was the most cost-effective strategy for managing cervical precancer in China. Additional triage approach with quality-assured performance could reduce the overtreatment and remains highly cost-effective in cervical cancer prevention. Screen-and-treat strategies would contribute to initiate screening and treatment services for the countries without national cervical cancer screening program rapidly, and meanwhile, scale up the screening and treatment coverage for the countries with the national program but have yet achieved 70% and 90% targets. The actions taking such strategies into the national program would promote health equity and accelerate the elimination of cervical cancer worldwide.

## Supplementary Information

**Additional file 1:** **Supplementary Materials. Fig. S1.** Model structure. **Fig. S2.** Model outputs from the 1000 Monte Carlo simulations. **Fig. S3a.** Process of screening strategy with self-HPV without triage. **Fig. S3b.** Process of screening strategy with self-HPV16/18 triage. **Fig. S3c.** Process of screening strategy with self-HPV7 types triage. **Fig. S3d.** Process of screening strategy with physician-HPV without triage. **Fig. S3e.** Process of screening strategy with physician-HPV16/18 triage. **Fig. S3f.** Process of screening strategy with physician-HPV7 types triage. **Fig. S3g.** Process of screening strategy with physician-HPV with genotype triage. **Fig. S3h.** Process of screening strategy with physician-HPV with cytology triage. **Fig. S4.** Follow-up of women after treatment. **Fig. S5a.** Tornado diagram analysis for self-HPV without triage versus current physician-HPV with cytology strategy in urban area. **Fig. S5b.** Tornado diagram analysis for self-HPV without triage versus current physician-HPV with cytology strategy in rural area. **Table S1.** Natural history and transition probabilities parameters of cervical cancer. **Table S2a.** Cervical cancer screening costs for self-HPV without triage strategy. **Table S2b.** Cervical cancer screening costs for self-HPV16/18 triage strategy. **Table S2c.** Cervical cancer screening costs for self-HPV7 types triage strategy. **Table S2d.** Cervical cancer screening costs for physician-HPV without triage strategy. **Table S2e.** Cervical cancer screening costs for physician-HPV16/18 triage strategy. **Table S2f.** Cervical cancer screening costs for physician-HPV7 types triage strategy. **Table S2g.** Cervical cancer screening costs for physician-HPV with genotype triage strategy. **Table S2h.** Cervical cancer screening costs for physician-HPV with cytology triage strategy. **Table S3.** Disease status at follow-up based on combined endpoints. **Table S4.** Utility of women before and after thermal ablation or LEEP treatment. **Table S5a.** Lifetime costs, effectiveness, and incremental cost-effectiveness for all screen-and-treat strategies versus the currently used strategies (Undiscounted). **Table S5b.** Lifetime costs, effectiveness, and incremental cost-effectiveness for all screen-and-treat strategies versus the currently used strategies (Discounted, 3%).

## Data Availability

Chinese Cancer Registry data used in this paper are held by the National Cancer Center of China. Data for the parameters in the model are held by the model research team. Access to the data can be requested by contacting the corresponding author. Information of population size data is freely downloadable from the National Bureau of Statistics of China.

## Abbreviations

ASCUS: Atypical squamous cell of undetermined significance
CIN: Cervical intraepithelial neoplasia
GDP: Gross domestic product
HPV: Human papillomaviruses
ICER: Cost-effectiveness ratios
LEEP: Loop electrosurgical excision procedure
LMICs: Low- and middle-income countries
PHC: Primary health care
QALYs: Quality-adjusted life-years
QOL: Quality-of-life
VIA: Visual inspection with acetic acid
WHO: World Health Organization
WTP: Willing to pay
