# Supplementary material for: Cost-effectiveness of the screen-and-treat strategies using HPV test linked to thermal ablation for cervical cancer prevention in China: a modeling study
Source: BMC Med. 2023 Apr 17;21:149. doi: 10.1186/s12916-023-02840-8 (PMC10111823; doi:10.1186/s12916-023-02840-8)
Supplement: Supplementary file 1 — Additional file 1: Supplementary Materials. Fig. S1. Model structure. Fig. S2. Model outputs from the 1000 Monte Carlo simulations. Fig. S3a. Process of screening strategy with self-HPV without triage. Fig. S3b. Process of screening strategy with self-HPV16/18 triage. Fig. S3c. Process of screening strategy with self-HPV7 types triage. Fig. S3d. Process of screening strategy with physician-HPV without triage. Fig. S3e. Process of screening strategy with physician-HPV16/18 triage. Fig. S3f. Process of screening strategy with physician-HPV7 types triage. Fig. S3g. Process of screening strategy with physician-HPV with genotype triage. Fig. S3h. Process of screening strategy with physician-HPV with cytology triage. Fig. S4. Follow-up of women after treatment. Fig. S5a. Tornado diagram analysis for self-HPV without triage versus current physician-HPV with cytology strategy in urban area. Fig. S5b. Tornado diagram analysis for self-HPV without triage versus current physician-HPV with cytology strategy in rural area. Table S1. Natural history and transition probabilities parameters of cervical cancer. Table S2a. Cervical cancer screening costs for self-HPV without triage strategy. Table S2b. Cervical cancer screening costs for self-HPV16/18 triage strategy. Table S2c. Cervical cancer screening costs for self-HPV7 types triage strategy. Table S2d. Cervical cancer screening costs for physician-HPV without triage strategy. Table S2e. Cervical cancer screening costs for physician-HPV16/18 triage strategy. Table S2f. Cervical cancer screening costs for physician-HPV7 types triage strategy. Table S2g. Cervical cancer screening costs for physician-HPV with genotype triage strategy. Table S2h. Cervical cancer screening costs for physician-HPV with cytology triage strategy. Table S3. Disease status at follow-up based on combined endpoints. Table S4. Utility of women before and after thermal ablation or LEEP treatment. Table S5a. Lifetime costs, effectiveness, and incremental cost-e [file 12916_2023_2840_MOESM1_ESM.docx]

**Cost-effectiveness of the screen and treat strategies using HPV test linked to thermal ablation for cervical cancer prevention in China: a modelling study**

**Additional File 1**: **Supplementary Materials**

Xue-Lian Zhao (#), MD; Shuang Zhao (#), MD; Chang-Fa Xia, MD; Shang-Ying Hu, MD; Xian-Zhi Duan, MD; Zhi-Hua Liu, MD; Yue-Yun Wang, MD; Ting-Ting You, MPH; Meng Gao, MPH; You-Lin Qiao, MD; Partha Basu, MD; Fang-Hui Zhao*,MD.

Contents

[1. Model 2](#_Toc56957307)

[*1.1 Model structure*](#_Toc56957314) *2*

[*1.2 Model validation*](#_Toc56957315) *3*

[2. Screening strategy](#_Toc56957313) 4

[*2.1 Scenarios*](#_Toc56957314) *4*

[*2.2 Follow up after thermal ablation or LEEP treatment 1*](#_Toc56957315)*3*

[3. Parameters 14](#_Toc56957324)

[*3.1 Natural history and transition probabilities parameters of cervical cancer 14*](#_Toc56957314)

[*3.2 Demographic and epidemiological data 15*](#_Toc56957314)

[*3.3 Characteristics of screening tests, treatment approach, screening program 16*](#_Toc56957314)

[*3.4 Cost data 17*](#_Toc56957315)

[*3.5 Utility data 22*](#_Toc56957321)

[4. Supplemental results 23](#_Toc56957325)

[*4.1 Lifetime costs, effectiveness, and incremental cost-effectiveness for all screen–and–treat strategies versus the currently used strategies 24*](#_Toc56957314)

[*4.2 Tornado diagram analysis for optimal strategy (self-HPV without triage) versus current strategy (physician-HPV with cytology) in urban area 26*](#_Toc56957314)

[*4.3 Tornado diagram analysis for optimal strategy (self-HPV without triage) versus current strategy (physician-HPV with cytology) in rural area 27*](#_Toc56957314)

[5. References 28](#_Toc56957325)

This appendix provides additional details on methods, assumptions, and results presented in the main manuscript. The hybrid model used in our analysis has been previously published [[1](#_ENREF_1),[2](#_ENREF_2)]. We summarize the adaptation and parameterization of the model below.

1. **Model**
   1. **Model structure**

The hybrid Model consisted of a dynamic model and a natural history model (Fig. S1). Dynamic model was used to simulate the human papillomavirus (HPV) transmission between males and females, whereas the natural history model was employed to simulate the natural history of cervical cancer and to obtain the number of cervical cancer cases and deaths associated with HPV infections. Details of the model development process, including initial parameterization and calibration, have been previously published [[1](#_ENREF_1),[2](#_ENREF_2)].

We simulated a cohort of 100,000 Chinese females, who entered into the model at their birth age. The women were screened as specified screening strategy during 30 to 65 years old with interval of 5 years. In this simulation, all women were followed throughout their lifetimes. Vaccination was not considered in the model.

**Fig. S1. Model structure**

HPV=human papillomavirus. CIN=cervical intraepithelial neoplasia. CC=cervical cancer. FOI=force of infection.

The hybrid model of HPV infection and cervical carcinogenesis followed a cohort of individual females from birth until death. The health states among individuals represented HPV infection, the grade of cervical intraepithelial neoplasia (grade 1, grades 2 and 3), and the stage of invasive cancer. Movement through the health states occurred in yearly increments according to probabilities that were dependent on age and HPV status. Transitions from the HPV infection to other health states were governed by genotype. Women with cervical cancer could be detected via symptoms or screening, and were subject to stage-specific mortality rates in addition to all-cause mortality rates. In this model, we updated the hybrid model which was suitable for cervical cancer screening strategies to be evaluated in this model analysis. Models were calibrated using epidemiological data of high-risk HPV prevalence, cervical cancer incidence and mortality in 2015, HPV type distributions in women with normal cervical cytology, low-grade cervical precancerous lesions, high-grade cervical precancerous lesions, and invasive cervical cancer [[3-7](#_ENREF_3)]. The results of model calibration have been represented in the previous published paper [[1](#_ENREF_1),[2](#_ENREF_2)]. The model tracked disease progression and regression, clinical events, and economic outcomes over the lifetime for all woman, and health outcomes and cost were then aggregated for analysis.

- 1. **Model validation**

Our model has been validated by comparing model predictions with observed data from a population-based cohort study in China[8]. We ran 1,000 Monte Carlo simulations to sample parameter values from their distributions and estimate three indicators: 1) prevalent risk of CIN2+ after one positive HPV test, 2) five-year cumulative risk of CIN2+ after one positive HPV test, and 3) 10-year cumulative detection rate of CIN2+outcomes. The results demonstrated good agreement between simulated and observed data (Fig. S2).


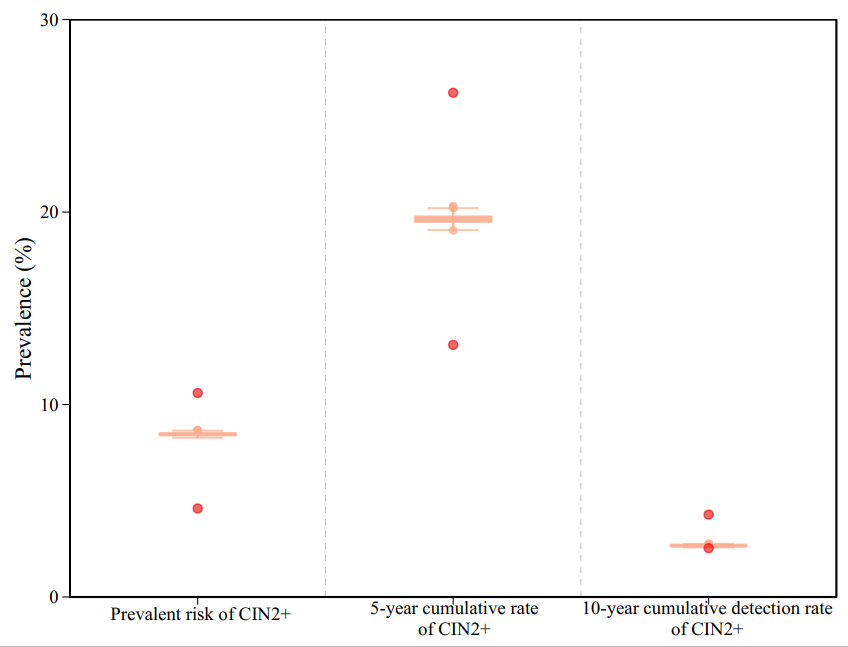


**Fig. S2. Model outputs from the 1000 Monte Carlo simulations**

Red points in vertical represent the 95% confidential interval reported in the population-based cohort study. Box plots indicate the distribution of results by running 1,000 Monte Carlo simulations.

1. **Screening strategy**

**2.1 Scenarios**

A total of six screen-and-treat strategies and two currently used screening strategies were simulated in this model analysis. Overall, the six alternative scenarios could be divided into *screen-and-treat* strategy (with treatment provided immediately following receipt of HPV positive result. Scenario1, 4), *screen, triage-and-treat* strategy (the decision to treat is based on a primary HPV positive followed by a positive “triage” test. Scenario 2, 3, 5, 6) and the current strategy (most commonly used in the present cervical cancer screening practice. Scenario7, 8). In each strategy, screening scenarios were stratified by physician-sampling or self-sampling, triage and treatment modalities. The simulated scenarios were as below.

Scenario 1: self-HPV without triage (Fig. S3a)

*Activities at primary health care facilities:* Participants collect cervical-vaginal samples by themselves at primary care centers.

*Activities at secondary health care facilities:* All samples are transferred and tested for 13 types of high-risk HPV (hrHPV) DNA (HPV16,18,31,33,35,39,45,51,52,56,58,59,68) with polymerase chain reaction (PCR)-based HPV test in laboratories of secondary health care facilities. Women with hrHPV positive result are invited for the visit in the secondary health care facilities. Among them, those eligible for thermal ablation (TA) are immediately treated with TA, and women not eligible for TA or suspected with cancer are taken biopsy if abnormal colposcopy. Women with histologically confirmed CIN2 or CIN3 are invited for loop electrosurgical excision procedure (LEEP) treatment.

*Activities at tertiary health care facilities:* women diagnosed with cervical cancer are suggested to receive treatment and further management in tertiary health care facilities.

*Follow-up:* Women with hrHPV negative results are routinely rescreened in five years. Women positive on hrHPV and with CIN1 or less were rescreened in one year. The follow-up procedure for women treated with TA or LEEP have been presented in the **part 2.2**.

**Fig. S3a. Process of screening strategy with self-HPV without triage**

hrHPV=high risk human papillomavirus. TA=thermal ablation. CIN=cervical intraepithelial neoplasia. LEEP=loop electrosurgical excision procedure.

Scenario 2: self-HPV16/18 triage (Fig. S3b)

The activities performed in primary and tertiary health care facilities, and the follow-up procedures are same with Scenario 1.

*Activities at secondary health care facilities:* All samples are transferred and tested for 13 types of hrHPV DNA (HPV16,18,31,33,35,39,45,51,52,56,58,59,68) with PCR-based HPV test in laboratories of secondary health care facilities. Women with hrHPV positive result are invited for the visit in the secondary health care facilities. Among them, women positive on HPV16/18 and eligible for TA are immediately treated; women positive on other types of hrHPV and eligible for TA are immediately if any CIN is suspected on colposcopy evaluation; and women not eligible for TA or suspected with cancer are taken biopsy if abnormal colposcopy. Women with histologically confirmed CIN2 or CIN3 are invited for LEEP treatment.

**Fig. S3b. Process of screening strategy with self-HPV16/18 triage**

hrHPV=high risk human papillomavirus. TA=thermal ablation. CIN=cervical intraepithelial neoplasia. LEEP=loop electrosurgical excision procedure.

Scenario 3: self-HPV7 types triage (Fig. S3c)

The activities performed in primary and tertiary health care facilities, and the follow-up procedures are same with Scenario 1.

*Activities at secondary health care facilities:* All samples are transferred and tested for 13 types of hrHPV DNA (HPV16,18,31,33,35,39,45,51,52,56,58,59,68) using PCR-based HPV test in laboratories of secondary health care facilities. Women with hrHPV positive result are invited for the visit in the secondary health care facilities. Among them, women positive on HPV16/18/31/33/45/52/58 and eligible for TA are immediately treated; women positive on other types of hrHPV and eligible for TA are immediately treated if any CIN is suspected on colposcopy evaluation; and women not eligible for TA or suspected with cancer are taken biopsy if abnormal colposcopy. Women with histologically confirmed CIN2 or CIN3 are invited for LEEP treatment.

**Fig. S3c. Process of screening strategy with self-HPV7 types triage**

hrHPV=high risk human papillomavirus. TA=thermal ablation. CIN=cervical intraepithelial neoplasia. LEEP=loop electrosurgical excision procedure.

Scenario 4: physician-HPV without triage (Fig. S3d)

*Activities at secondary health care facilities:* Participants are collected cervical samples by physicians at secondary health care facilities. All samples are tested for 13 types of hrHPV DNA (HPV16,18,31,33,35,39,45,51,52,56,58,59,68) by point-of-care PCR-based HPV test (Rapid testing, allowing participants to receive HPV test results and proceed to the next step at the same visit) in laboratories of secondary health care facilities. In the same visit with the HPV test, women positive on hrHPV and eligible for TA are immediately treated in the same visit; women positive on hrHPV but not eligible for TA or suspected with cancer are taken biopsy if abnormal colposcopy. Women with histologically confirmed CIN2 or CIN3 are invited for LEEP treatment in another visit.

The activities performed in tertiary health care facilities and the follow-up procedures are same with Scenario 1.

**Fig. S3d. Process of screening strategy with physician-HPV without triage**

hrHPV=high risk human papillomavirus. TA=thermal ablation. CIN=cervical intraepithelial neoplasia. LEEP=loop electrosurgical excision procedure.

Scenario 5: physician-HPV16/18 triage (Fig. S3e)

*Activities at secondary health care facilities:* Participants are collected cervical samples by physicians at secondary health care facilities. All samples are tested for 13 types of hrHPV DNA (HPV16,18,31,33,35,39,45,51,52,56,58,59,68) by point-of-care PCR-based HPV test (Rapid testing, allowing participants to receive HPV test results and proceed to the next step at the same visit) in laboratories of secondary health care facilities. In the same visit with the HPV test, women positive on HPV16/18 and eligible for TA are immediately treated in the same visit; women positive on other types of hrHPV and eligible for TA are immediately treated if any CIN is suspected on colposcopy evaluation; and women positive on hrHPV but not eligible for TA or suspected with cancer are taken biopsy if abnormal colposcopy. Women with histologically confirmed CIN2 or CIN3 are invited for LEEP treatment in another visit.

The activities performed in tertiary health care facilities and the follow-up procedures are same with Scenario 1.

**Fig. S3e. Process of screening strategy with physician-HPV16/18 triage**

hrHPV=high risk human papillomavirus. TA=thermal ablation. CIN=cervical intraepithelial neoplasia. LEEP=loop electrosurgical excision procedure.

Scenario 6: physician-HPV7 types triage (Fig. S3f)

*Activities at secondary health care facilities:* Participants are collected cervical samples by physicians at secondary health care facilities. All samples are tested for 13 types of hrHPV DNA (HPV16,18,31,33,35,39,45,51,52,56,58,59,68) by point-of-care PCR-based HPV test (Rapid testing, allowing participants to receive HPV test results and proceed to the next step at the same visit) in laboratories of secondary health care facilities. In the same visit with the HPV test, women positive on HPV16/18/31/33/45/52/58 and eligible for TA are immediately treated in the same visit; women positive on other types of hrHPV and eligible for TA are immediately treated if any CIN is suspected on colposcopy evaluation; and women positive on hrHPV but not eligible for TA or suspected with cancer are taken biopsy if abnormal colposcopy. Women with histologically confirmed CIN2 or CIN3 are invited for LEEP treatment in another visit.

The activities performed in tertiary health care facilities and the follow-up procedures are same with Scenario 1.

**Fig. S3f. Process of screening strategy with physician-HPV7 types triage**

hrHPV=high risk human papillomavirus. TA=thermal ablation. CIN=cervical intraepithelial neoplasia. LEEP=loop electrosurgical excision procedure.

***Current strategies (most commonly used in the present practice)***

Scenario 7: physician-HPV with genotype triage (Fig. S3g)

*Activities at secondary health care facilities:* Participants are collected cervical samples by physicians at secondary health care facilities in the first visit. All samples are tested for 13 types of hrHPV DNA (HPV16,18,31,33,35,39,45,51,52,56,58,59,68) by PCR-based HPV test in laboratories of secondary health care facilities. Samples positive on hrHPV types other than HPV16/18 are tested by liquid-based cytology (LBC) as well. In the second visit, women positive on hrHPV 16/18, or other hrHPV types combined with atypical squamous cells of undetermined significance or worse (ASCUS+) are invited for colposcopy and biopsy if abnormal colposcopy. Women with histologically confirmed CIN2 or CIN3 are invited for LEEP treatment in the third visit.

The activities performed in tertiary health care facilities and the follow-up procedures are same with Scenario 1.

**Fig. S3g. Process of screening strategy with physician-HPV with genotype triage**

hrHPV=high risk human papillomavirus. LBC=liquid-based cytology. ASCUS+= atypical squamous cells of undetermined significance or worse. CIN=cervical intraepithelial neoplasia. LEEP=loop electrosurgical excision procedure.

Scenario 8: physician-HPV with cytology triage (Fig. S3h)

*Activities at secondary health care facilities:* Participants are collected cervical samples by physicians at secondary health care facilities in the first visit. All samples are tested for 13 types of hrHPV DNA (HPV16,18,31,33,35,39,45,51,52,56,58,59,68) by PCR-based HPV test in laboratories of secondary health care facilities. Samples positive on hrHPV types are tested by LBC as well. In the second visit, women with ASCUS+ are invited for colposcopy and biopsy if abnormal colposcopy. Women with histologically confirmed CIN2 or CIN3 are invited for LEEP treatment in the third visit.

The activities performed in tertiary health care facilities and the follow-up procedures are same with Scenario 1.

**Fig.** **S3h. Process of screening strategy with physician-HPV with cytology triage**

hrHPV=high risk human papillomavirus. LBC=liquid-based cytology. ASCUS+=atypical squamous cells of undetermined significance or worse. CIN=cervical intraepithelial neoplasia. LEEP=loop electrosurgical excision procedure.

**2.2 Follow up after thermal ablation or LEEP treatment**

All treated women would be followed up in 12 months after treatment by using HPV tests (follow the initial sampling method of the strategies) in the secondary facilities. Women negative on HPV test are rescreened in 24 months after treatment. Women positive on HPV test are referred to colposcopy. And gynecologist undertook biopsies if abnormal colposcopy. Women with histologically confirmed CIN2 or CIN3 are invited for LEEP treatment. Women diagnosed with cervical cancer are suggested to receive treatment and further management in the tertiary health care facilities. Women positive on HPV and with CIN1 or less are rescreened in the next year (Fig. S4).

**Fig. S4. Follow-up of women after treatment**

^#^Women negative on HPV test are allowed to return to routine screening

HPV=human papillomavirus. TA=thermal ablation. CIN=cervical intraepithelial neoplasia. LEEP=loop electrosurgical excision procedure.

1. **Parameters**

Parameters presented here consists of 1) parameters associated with natural history and transition probabilities of cervical cancer; 2) demographic and epidemiological data; 3) characteristics of screening tests, treatment approach, screening program; 4) cost data of screening and treatment; 5) utility and quality of life.

- 1. **Parameters related to natural history and transition probabilities of cervical cancer**

Parameters related to natural history and transition probabilities of cervical cancer were extracted from open-source publications and were shown on Table S1.

**Table S1. Natural history and transition probabilities parameters of cervical cancer**

| Parameter | Base case | Range | Distribution | Source |
| --- | --- | --- | --- | --- |
| Transition probabilities |  |  |  |  |
| HPV clearance |  |  |  |  |
| 12–24 years | 0.7188 | 0.6463–0.7830 | β | [[9-13](#_ENREF_8)] |
| 25–29 years | 0.6984 | 0.5898–0.7952 | β | [[9-13](#_ENREF_8)] |
| 30–39 years | 0.3503 | 0.2860–0.4188 | β | [[9-13](#_ENREF_8)] |
| 40–49 years | 0.2048 | 0.1118–0.3022 | β | [[9-13](#_ENREF_8)] |
| >50 years | 0.1004 | 0.0546–0.1567 | β | [[9-13](#_ENREF_8)] |
| Progression and regression |  |  |  |  |
| HPV to CIN1 | 0.0717  (0.2/36 months) | 0.0527–0.1121  (0.15–0.30/36months) | β | [[12](#_ENREF_11),[15](#_ENREF_14)] |
| CIN1 to CIN2 | 0.2240 | 0.1608–0.2972 | β | [[9-13](#_ENREF_8)] |
| CIN2 to CIN3 | 0.3498 | 0.2623–0.4372 | β | [[9-13](#_ENREF_8)] |
| CIN3 to Cancer | 0.1019 | 0.0764–0.1274 | β | [[9-13](#_ENREF_8)] |
| CIN1 to HPV | 0.7008 | 0.6077–0.7933 | β | [[9-13](#_ENREF_8)] |
| CIN2 to CIN1 | 0.2494 | 0.1994–0.2992 | β | [[9-13](#_ENREF_8)] |
| CIN3 to CIN2 | 0.0135 | ±25% | β | [[12](#_ENREF_11)] |
| HPV to CIN2 | 0.0115 | 0.0034–0.0234 | β | [[9-13](#_ENREF_8)] |
| CIN2 to HPV | 0.1901 | ±25% | β | [[12](#_ENREF_11),[16](#_ENREF_15)] |
| CIN1 to CIN3 | 0.0464 | 0.0098–0.1297 | β | [[9-13](#_ENREF_8)] |
| aCCⅠ to aCC Ⅱ | 0.4377 (0.9/4 years) | ±25% | β | [[15](#_ENREF_14),[17](#_ENREF_16)] |
| aCCⅡ to aCC Ⅲ | 0.5358 (0.9/3years) | ±25% | β | [[15](#_ENREF_14),[17](#_ENREF_16)] |
| aCC Ⅲ to aCC Ⅳ | 0.6838 (0.9/2years) | ±25% | β | [[15](#_ENREF_14),[17](#_ENREF_16)] |
| Cancer symptoms |  |  |  |  |
| CC FIGO Ⅰ | 0.15 | ±25% | β | [[15](#_ENREF_14),[17](#_ENREF_16)] |
| CC FIGO Ⅱ | 0.225 | ±25% | β | [[15](#_ENREF_14),[17](#_ENREF_16)] |
| CC FIGO Ⅲ | 0.6 | ±25% | β | [[15](#_ENREF_14),[17](#_ENREF_16)] |
| CC FIGO Ⅳ | 0.9 | ±25% | β | [[15](#_ENREF_14),[17](#_ENREF_16)] |

HPV=human papillomavirus. CIN=cervical intraepithelial neoplasia. CC=cervical

cancer. aCC=asymptomatic cervical cancer. FIGO=International Federation of Gynecology and

Obstetrics.

- 1. **Demographic and epidemiological data**

Demographic and epidemiological data were extracted from open-source publications or online datasets published by the government. Information of population size in 2015 was derived from National Bureau of Statistics of China [[4](#_ENREF_4),[7](#_ENREF_7)]. All-cause mortality data was derived from China Health Statistics Year book released by National Health Commission of China [[5](#_ENREF_5)]. Cervical cancer incidence and mortality data was derived from Chinese Cancer Registry Report published by National Cancer Center of China [[6](#_ENREF_6)].

**3.3 Characteristics of screening tests, treatment approach, screening program**

***1) Efficacy of screening and treatment strategies***

The sensitivity and specificity of screen-and-treat strategies using self-sampling HPV DNA test are based on large-scale population-based studies in China [[18](#_ENREF_17)]. This is a prospective study which recruited 9526 women from three rural sites (Etuoke County in Inner Mongolia, Xiangyuan and Yangcheng counties in Shanxi Province) in 2017, with aim to validate a novel screening strategy (self-sampling HPV test combined with immediately thermal ablation treatment) in rural China. The sensitivity and specificity of strategies using physician-sampling are derived from the previous published articles [[19](#_ENREF_18),[20](#_ENREF_19)]. The efficacy of thermal ablation and LEEP treatment is collected from open-source publications [[21](#_ENREF_20),[22](#_ENREF_21)].

***2) Characteristics of screening program***

The 2030 targets have set the cervical cancer screening target at 70% coverage. Therefore, we assumed that the anticipated screening coverage would reach 70% with physician-collected samples, and 89.6% (95% CI 63.0 to 100.0%) with self-collected samples according to the ratio of screening uptake reported in a meta-analysis (self-sampling participation versus physician-sampling, RR: 1.28, 95% CI 0.90 to 1.82) [[23](#_ENREF_22)].

The rate of loss to follow-up impact cost accrual in the microsimulation model. We assumed that the rate of loss to follow-up would increase by 15% for each additional visit and varied this increase from 0 to 50% in the sensitivity analysis [[24](#_ENREF_23)].

***3) Characteristics of precancer management and treatment***

The proportions of women who were eligible and treated with thermal ablation stratified by the disease status were estimated according to our large-scale population-based studies in China [[18](#_ENREF_17)]. Meanwhile, the eligibility rates for thermal ablation was standardized according to the age structure of China's National Population Census in 2010 [[25](#_ENREF_24)]. For current screening strategies, the rates of CIN1 management and CIN2/CIN3 treatment refers to previous open-source publications[[1](#_ENREF_1),[2](#_ENREF_2)].

**3.4 Cost data**

***1) Screening and diagnosis cost***

A total of six screen-and-treat strategies and two currently used strategies were simulated in our model analysis. Considering the constantly declining price of HPV tests in China, the unit price of government procurement in many regions is below 40 CNY [[26-28](#_ENREF_25)], the price of the materials for the HPV test would be the same as the price of the *care*HPV test, which is 35 CNY. HPV tests related equipment costs, personnel costs, non-medical costs, and indirect cost were estimated based on our previous nationwide cervical cancer screening demonstration research, and the prices have been converted to that in 2020 [[29](#_ENREF_28)]. For self-sampling strategies, the specimens were transferred to the laboratory on ice by weekly, that is about 100-150 per week. The costs of transportation were respectively 70 and 50 CNY in urban and rural areas referring to the charging standard of cold chain logistics in China. Therefore, the unite price of specimen transfer is estimated as 0.7 and 0.5 CNY in urban and rural areas, respectively. Consistent with cost calculation table provided by government and our population-based pooled data, we estimate that 15% of women will be detected positive for hrHPV infection and 4% will be detected positive for HPV16/18 infection [[3](#_ENREF_3),30]. Cervical cancer screening costs (Chinese yuan, CNY) for each strategy are shown in Table S2a – S2h.

**Table S2a. Cervical cancer screening costs for self-HPV without triage strategy**

| Subjects | | Urban area | |  | Rural area | |
| --- | --- | --- | --- | --- | --- | --- |
|  | | Unit price | Subtotal |  | Unit price | Subtotal |
| HPV test | Materials | 35 | 35 |  | 35 | 35 |
|  | Equipment [[29](#_ENREF_28)] | 0.5 | 0.5 |  | 0.5 | 0.5 |
|  | Personnel costs [[29](#_ENREF_28),[31](#_ENREF_30)] | 5 | 5 |  | 2 | 2 |
|  | Transfer of specimen | 0.7 | 0.7 |  | 0.5 | 0.5 |
|  | Indirect cost  [[2](#_ENREF_2),[29](#_ENREF_28)] | Direct cost*25% | 10.3 |  | Direct cost*25% | 9.5 |
| Colposcopy (15.00% from HPV test) | | 60 | 9 |  | 60 | 9 |
| Medical service fee of colposcopy referral | | 10 | 1.5 |  | 10 | 1.5 |
| Histopathology (27.86% from colposcopy) | | 160 | 6.69 |  | 160 | 6.69 |
| Total | CNY (Chinese yuan) |  | 68.69 |  |  | 64.69 |
|  | USD (United States dollar) |  | 9.81 |  |  | 9.24 |

**Table S2b. Cervical cancer screening costs for self-HPV16/18 triage strategy**

| Subjects | | Urban area | |  | Rural area | |
| --- | --- | --- | --- | --- | --- | --- |
|  | | Unit price | Subtotal |  | Unit price | Subtotal |
| HPV test | Materials | 35 | 35 |  | 35 | 35 |
|  | Equipment [[29](#_ENREF_28)] | 0.5 | 0.5 |  | 0.5 | 0.5 |
|  | Personnel costs [[29](#_ENREF_28),[31](#_ENREF_30)] | 5 | 5 |  | 2 | 2 |
|  | Transfer of specimen | 0.7 | 0.7 |  | 0.5 | 0.5 |
|  | Indirect cost[[2](#_ENREF_2),[29](#_ENREF_28)] | Direct cost*25% | 10.3 |  | Direct cost*25% | 9.5 |
| Colposcopy (15.00% from HPV test) | | 60 | 9 |  | 60 | 9 |
| Medical service fee of colposcopy referral | | 10 | 1.5 |  | 10 | 1.5 |
| Histopathology (21.74% from colposcopy) | | 160 | 5.22 |  | 160 | 5.22 |
| Total | CNY (Chinese yuan) |  | 67.22 |  |  | 63.22 |
|  | USD (United States dollar) |  | 9.60 |  |  | 9.03 |

**Table S2c. Cervical cancer screening costs for self-HPV7 types triage strategy**

| Subjects | | Urban area | |  | Rural area | |
| --- | --- | --- | --- | --- | --- | --- |
|  | | Unit price | Subtotal |  | Unit price | Subtotal |
| HPV test | Materials | 35 | 35 |  | 35 | 35 |
|  | Equipment [[29](#_ENREF_28)] | 0.5 | 0.5 |  | 0.5 | 0.5 |
|  | Personnel costs [[29](#_ENREF_28),[31](#_ENREF_30)] | 5 | 5 |  | 2 | 2 |
|  | Transfer of specimen | 0.7 | 0.7 |  | 0.5 | 0.5 |
|  | Indirect cost [[2](#_ENREF_2),[29](#_ENREF_28)] | Direct cost*25% | 10.3 |  | Direct cost*25% | 9.5 |
| Colposcopy (15.00% from HPV test) | | 60 | 9 |  | 60 | 9 |
| Medical service fee of colposcopy referral | | 10 | 1.5 |  | 10 | 1.5 |
| Histopathology (20.41% from colposcopy) | | 160 | 4.90 |  | 160 | 4.90 |
| Total | CNY (Chinese yuan) |  | 66.90 |  |  | 62.90 |
|  | USD (United States dollar) |  | 9.56 |  |  | 8.99 |

**Table S2d. Cervical cancer screening costs for physician-HPV without triage strategy**

| Subjects | | Urban area | |  | Rural area | |
| --- | --- | --- | --- | --- | --- | --- |
|  | | Unit price | Subtotal |  | Unit price | Subtotal |
| Gynecological examination | | 15 | 15 |  | 15 | 15 |
| HPV test | Materials | 35 | 35 |  | 35 | 35 |
|  | Equipment [[29](#_ENREF_28)] | 0.5 | 0.5 |  | 0.5 | 0.5 |
|  | Personnel costs [[29](#_ENREF_28),[31](#_ENREF_30)] | 15 | 15 |  | 6 | 6 |
|  | Non-medical costs | 5 | 5 |  | 2 | 2 |
|  | Indirect cost [[2](#_ENREF_2),[29](#_ENREF_28)] | Direct cost*25% | 13.88 |  | Direct cost*25% | 10.88 |
| Colposcopy (15.00% from HPV test) | | 60 | 9 |  | 60 | 9 |
| Medical service fee of colposcopy referral | | 10 | 1.5 |  | 10 | 1.5 |
| Histopathology (27.86% from colposcopy) | | 160 | 6.69 |  | 160 | 6.69 |
| Total | CNY (Chinese yuan) |  | 101.57 |  |  | 86.57 |
|  | USD (United States dollar) |  | 14.51 |  |  | 12.37 |

**Table S2e. Cervical cancer screening costs for physician-HPV16/18 triage strategy**

| Subjects | | Urban area | |  | Rural area | |
| --- | --- | --- | --- | --- | --- | --- |
|  | | Unit price | Subtotal |  | Unit price | Subtotal |
| Gynecological examination | | 15 | 15 |  | 15 | 15 |
| HPV test | Materials | 35 | 35 |  | 35 | 35 |
|  | Equipment [[29](#_ENREF_28)] | 0.5 | 0.5 |  | 0.5 | 0.5 |
|  | Personnel costs [[29](#_ENREF_28),[31](#_ENREF_30)] | 15 | 15 |  | 6 | 6 |
|  | Non-medical costs | 5 | 5 |  | 2 | 2 |
|  | Indirect cost [[2](#_ENREF_2),[29](#_ENREF_28)] | Direct cost*25% | 13.88 |  | Direct cost*25% | 10.88 |
| Colposcopy (15.00% from HPV test) | | 60 | 9 |  | 60 | 9 |
| Medical service fee of colposcopy referral | | 10 | 1.5 |  | 10 | 1.5 |
| Histopathology (21.74% from colposcopy) | | 160 | 5.22 |  | 160 | 5.22 |
| Total | CNY (Chinese yuan) |  | 100.10 |  |  | 85.10 |
|  | USD (United States dollar) |  | 14.30 |  |  | 12.16 |

**Table S2f. Cervical cancer screening costs for physician-HPV7 types triage strategy**

| Subjects | | Urban area | |  | Rural area | |
| --- | --- | --- | --- | --- | --- | --- |
|  | | Unit price | Subtotal |  | Unit price | Subtotal |
| Gynecological examination | | 15 | 15 |  | 15 | 15 |
| HPV test | Materials | 35 | 35 |  | 35 | 35 |
|  | Equipment [[29](#_ENREF_28)] | 0.5 | 0.5 |  | 0.5 | 0.5 |
|  | Personnel costs [[29](#_ENREF_28),[31](#_ENREF_30)] | 15 | 15 |  | 6 | 6 |
|  | Non-medical costs | 5 | 5 |  | 2 | 2 |
|  | Indirect cost [[2](#_ENREF_2),[29](#_ENREF_28)] | Direct cost*25% | 13.88 |  | Direct cost*25% | 10.88 |
| Colposcopy (15.00% from HPV test) | | 60 | 9 |  | 60 | 9 |
| Medical service fee of colposcopy referral | | 10 | 1.5 |  | 10 | 1.5 |
| Histopathology (20.41% from colposcopy) | | 160 | 4.90 |  | 160 | 4.90 |
| Total | CNY (Chinese yuan) |  | 99.78 |  |  | 84.78 |
|  | USD (United States dollar) |  | 14.25 |  |  | 12.11 |

**Table S2g. Cervical cancer screening costs for physician-HPV with genotype triage strategy**

| Subjects | | Urban area | |  | Rural area | |
| --- | --- | --- | --- | --- | --- | --- |
|  | | Unit price | Subtotal |  | Unit price | Subtotal |
| Gynecological examination | | 15 | 15 |  | 15 | 15 |
| HPV test | Materials | 35 | 35 |  | 35 | 35 |
|  | Equipment [[29](#_ENREF_28)] | 0.5 | 0.5 |  | 0.5 | 0.5 |
|  | Personnel costs [[29](#_ENREF_28),[31](#_ENREF_30)] | 15 | 15 |  | 6 | 6 |
|  | Non-medical costs | 5 | 5 |  | 2 | 2 |
|  | Indirect cost[[2](#_ENREF_2),[29](#_ENREF_28)] | Direct cost*25% | 13.88 |  | Direct cost*25% | 10.88 |
| LBC (11.00% from HPV test) | | 66 | 7.26 |  | 66 | 7.26 |
| Colposcopy (49.04% from LBC and 4.00% from HPV test) | | 60 | 5.64 |  | 60 | 5.64 |
| Medical service fee of colposcopy referral | | 10 | 0.94 |  | 10 | 0.94 |
| Histopathology (34.04% from colposcopy) | | 160 | 5.12 |  | 160 | 5.12 |
| Total | CNY (Chinese yuan) |  | 103.33 |  |  | 88.33 |
|  | USD (United States dollar) |  | 14.76 |  |  | 12.62 |

**Table S2h. Cervical cancer screening costs for physician-HPV with cytology triage strategy**

| Subjects | | Urban area | |  | Rural area | |
| --- | --- | --- | --- | --- | --- | --- |
|  | | Unit price | Subtotal |  | Unit price | Subtotal |
| Gynecological examination | | 15 | 15 |  | 15 | 15 |
| HPV test | Materials | 35 | 35 |  | 35 | 35 |
|  | Equipment [[29](#_ENREF_28)] | 0.5 | 0.5 |  | 0.5 | 0.5 |
|  | Personnel costs [[29](#_ENREF_28),[31](#_ENREF_30)] | 15 | 15 |  | 6 | 6 |
|  | Non-medical costs | 5 | 5 |  | 2 | 2 |
|  | Indirect cost [[2](#_ENREF_2),[29](#_ENREF_28)] | Direct cost*25% | 13.88 |  | Direct cost*25% | 10.88 |
| LBC (15.00% from HPV test) | | 66 | 9.9 |  | 66 | 9.9 |
| Colposcopy (48.44% from LBC) | | 60 | 4.36 |  | 60 | 4.36 |
| Medical service fee of colposcopy referral | | 10 | 0.73 |  | 10 | 0.73 |
| Histopathology (32.21% from colposcopy) | | 160 | 3.74 |  | 160 | 3.74 |
| Total | CNY (Chinese yuan) |  | 103.11 |  |  | 88.11 |
|  | USD (United States dollar) |  | 14.73 |  |  | 12.59 |

***2) Treatment and follow-up costs for precancerous lesions***

***Treatment costs***

The cost of thermal ablation treatment was calculated using micro-costing approach considering the cost and life span of the equipment as well as the personal cost for each treatment, due to it has not been introduced to the clinical practice in China. The cost of the battery-driven ablator (Liger Medical LLC, Utah) is 9821.7 CNY ($1500). By assuming that 500 women could be treated within the equipment’s life span, the per capita cost of the thermal ablation treatment machine is 20 CNY. Personal cost is estimated as 60 CNY according to the cost of colposcopy examination due to the thermal ablation treatment is conducted under colposcopy. Therefore, the per capita cost of thermal ablation treatment is 80 CNY. In this model analysis, LEEP was considered as an outpatient procedure conducted under local anesthesia. The cost of LEEP treatment was derived from the average charges in the secondary facilities in China. The direct non-medical cost mainly refers to the round-trip transportation cost for women to receive treatment. Round-trip transportation costs at each level of health facility were based on the charging standard of the local public transportation, namely 2 CNY in rural areas and 5 CNY in urban areas. Indirect cost is estimated as 25% of direct cost [[2](#_ENREF_2),[29](#_ENREF_28)].

Costs associated with cancer care by stage (FIGO I-IIa or FIGO IIb-IV), including direct medical cost, direct non-medical costs and indirect costs, were derived from previous published paper [[2](#_ENREF_2),[29](#_ENREF_28),[32](#_ENREF_31),[33](#_ENREF_32)]. Costs were reported in **Table 2** of the main manuscript.

***Follow-up costs***

The unite costs for follow-up after thermal ablation or LEEP treatment were estimated by the average values of the total costs spent on HPV tests, colposcopy and histopathology diagnosis for treated women with HPV infection only, CIN1+, or CIN2/CIN3. The proportion of different disease status during the follow-up referred to the published literature and local population-based studies in China (Table S3) [[21](#_ENREF_20)].

**Table S3.** **Disease status at follow-up based on combined endpoints**

|  | Baseline histology before treatment | Follow up | | |
| --- | --- | --- | --- | --- |
|  |  | **Negative for HPV tests** | **Positive for HPV tests but normal on colposcopy** | **Positive for HPV tests and abnormal on colposcopy** |
| Thermal ablation | HPV positive | 81.4% | 14.5% | 4.2% |
|  | CIN1/CIN2/CIN3 | 67.7% | 18.1% | 14.2% |
| LEEP | CIN2/CIN3 | 68.4% | 18.4% | 13.2% |

**3.5 Utility data**

Utility scores for women treated with thermal ablation or LEEP were obtained from the quality-of-life (QOF) assessments in Chinese patients based on a multicenter survey, which was conducted in both rural (Etuoke County in Inner Mongolia, Xiangyuan and Yangcheng counties in Shanxi Province) and urban settings (Shenzhen in Guangdong province) in 2021 [34]. Chinese version of EQ-5D-5L (EuroQoL-5 Dimensions-5 Levels, EQ-5D-5L) scale were used to conduct the survey. MULT8 model (8-parameter multiplicative model) based on EQ-5D-5L value set for Chinese population was used to convert health status to utility [[35](#_ENREF_33)]. And utility scores were stratified by pre-treatment and 1 month post-treatment. The detail results are shown in Table S4.

**Table S4. Utility of women before and after thermal ablation or LEEP treatment**

|  | LEEP (n_1_ = 102)  Utility base case, (95%CI) | Thermal ablation (n_2_ = 93)  Utility base case, (95%CI) | *P value ^a^* |
| --- | --- | --- | --- |
| Pretreatment | 0.984(0.977-0.992) | 0.986(0.978-0.994) | 0.748 |
| 1 month posttreatment | 0.956(0.938-0.974) | 0.984(0.976-0.993) | 0.005 |
| *P value ^b^* | 0.002 | 0.515 |  |

^a^ Independent Samples T-Test (between groups)

^b^ Paired Samples T-Test (within group)

**4. Supplemental results**

Table S5a and Table S5b displayed lifetime costs, effectiveness, and incremental cost-effectiveness for all screen-and-treat strategies versus the currently used strategies.

Fig. S5a and Fig. S5b presented tornado diagram analysis for optimal strategy (self-HPV without triage) versus current strategy (physician-HPV with cytology) in urban area and rural China. In this diagram, each bar represented the impact of uncertainty in an individual variable on the ICER.

**Table S5a. Lifetime costs, effectiveness, and incremental cost-effectiveness for all screen-and-treat strategies versus the currently used strategies (Undiscounted)**

| Strategies | Total costs (million) | Total QALYs (million) | Strategy 7 as reference | | |  | Strategy 8 as reference | | | | |
| --- | --- | --- | --- | --- | --- | --- | --- | --- | --- | --- | --- |
|  |  |  | Incremental costs (million) | Incremental QALYs  (million) | ICER ($/QALY) |  | Incremental costs (million) | Incremental QALYs  (million) | | ICER ($/QALY) | |
| China |  | | | | | | | |  | |  |
| 1(self-HPV without triage) | 12.20724 | 7.86105 | -1.29527 | 0.00144 | -901.50 |  | -2.51734 | 0.00253 | | -997.10 | |
| 2(self-HPV16/18triage) | 13.00226 | 7.85988 | -0.50025 | 0.00027 | -1864.64 |  | -1.72232 | 0.00136 | | -1269.99 | |
| 3(self-HPV7 types triage) | 12.34086 | 7.86071 | -1.16165 | 0.00110 | -1055.96 |  | -2.38372 | 0.00219 | | -1089.46 | |
| 4(phy-HPV without triage) | 13.45400 | 7.86084 | -0.04851 | 0.00123 | -39.60 |  | -1.27058 | 0.00232 | | -549.34 | |
| 5(phy-HPV16/18 triage) | 14.07233 | 7.85972 | 0.56982 | 0.00011 | 5040.46 |  | -0.65225 | 0.00120 | | -543.11 | |
| 6(phy-HPV7 types triage) | 13.52594 | 7.86054 | 0.02343 | 0.00093 | 25.32 |  | -1.19864 | 0.00202 | | -595.28 | |
| 7(phy-HPV with genotype triage) | 13.50251 | 7.85961 | / | / | / |  | -1.22207 | 0.00109 | | -1123.35 | |
| 8(phy-HPV with cytology triage) | 14.72458 | 7.85852 | 1.22207 | -0.00109 | -1123.35 |  | / | / | | / | |
| Urban areas |  | | | | | | | | | |  |
| 1(self-HPV without triage) | 12.96306 | 7.89598 | -1.91263 | 0.00151 | -1263.62 |  | -3.36576 | 0.00264 | | -1273.80 | |
| 2(self-HPV16/18triage) | 14.04531 | 7.89476 | -0.83038 | 0.00030 | -2769.02 |  | -2.28351 | 0.00143 | | -1598.47 | |
| 3(self-HPV7 types triage) | 13.18434 | 7.89563 | -1.69135 | 0.00116 | -1453.06 |  | -3.14448 | 0.00229 | | -1371.53 | |
| 4(phy-HPV without triage) | 14.58693 | 7.89576 | -0.28876 | 0.00130 | -222.53 |  | -1.74190 | 0.00243 | | -717.91 | |
| 5(phy-HPV16/18 triage) | 15.45437 | 7.89461 | 0.57868 | 0.00015 | 3916.77 |  | -0.87445 | 0.00128 | | -685.08 | |
| 6(phy-HPV7 types triage) | 14.73099 | 7.89545 | -0.14470 | 0.00099 | -146.70 |  | -1.59783 | 0.00212 | | -755.45 | |
| 7(phy-HPV with genotype triage) | 14.87569 | 7.89446 | / | / | / |  | -1.45313 | 0.00113 | | -1287.46 | |
| 8(phy-HPV with cytology triage) | 16.32882 | 7.89334 | 1.45313 | -0.00113 | -1287.46 |  | / | / | | / | |
| Rural areas |  | | | | | | | | | |  |
| 1(self-HPV without triage) | 11.12537 | 7.81105 | -0.41159 | 0.00133 | -310.21 |  | -1.30293 | 0.00236 | | -552.95 | |
| 2(self-HPV16/18triage) | 11.50925 | 7.80995 | -0.02770 | 0.00022 | -124.20 |  | -0.91905 | 0.00125 | | -733.75 | |
| 3(self-HPV7 types triage) | 11.13351 | 7.81073 | -0.40345 | 0.00101 | -400.00 |  | -1.29479 | 0.00204 | | -635.29 | |
| 4(phy-HPV without triage) | 11.83234 | 7.81084 | 0.29538 | 0.00112 | 263.48 |  | -0.59596 | 0.00215 | | -277.12 | |
| 5(phy-HPV16/18 triage) | 12.09411 | 7.80979 | 0.55716 | 0.00006 | 8789.15 |  | -0.33419 | 0.00109 | | -305.79 | |
| 6(phy-HPV7 types triage) | 11.80107 | 7.81056 | 0.26411 | 0.00084 | 314.87 |  | -0.62724 | 0.00187 | | -335.73 | |
| 7(phy-HPV with genotype triage) | 11.53696 | 7.80972 | / | / | / |  | -0.89135 | 0.00103 | | -865.81 | |
| 8(phy-HPV with cytology triage) | 12.42830 | 7.80869 | 0.89135 | -0.00103 | -865.81 |  | / | / | | / | |

**Table S5b****. Lifetime costs, effectiveness, and incremental cost-effectiveness for all screen-and-treat strategies versus the currently used strategies (Discounted, 3%)**

| Strategies | Total  costs (million) | Total QALYs (million) | Strategy 7 as reference | | |  | Strategy 8 as reference | | |
| --- | --- | --- | --- | --- | --- | --- | --- | --- | --- |
|  |  |  | Incremental  costs  (million) | Incremental QALYs  (million) | ICER ($/QALY) |  | Incremental  costs  (million) | Incremental QALYs  (million) | ICER ($/QALY) |
| China |  |  |  |  |  |  |  |  |  |
| 1(self-HPV without triage) | 3.58311 | 3.66001 | -0.29589 | 0.00024 | -1238.98 |  | -0.58629 | 0.00042 | -1401.73 |
| 2(self-HPV16/18triage) | 3.73237 | 3.65982 | -0.14663 | 0.00005 | -3214.10 |  | -0.43703 | 0.00023 | -1941.82 |
| 3(self-HPV7 types triage) | 3.59944 | 3.65996 | -0.27956 | 0.00018 | -1527.90 |  | -0.56996 | 0.00036 | -1572.68 |
| 4(phy-HPV without triage) | 3.95903 | 3.65998 | 0.08003 | 0.00020 | 393.47 |  | -0.21037 | 0.00038 | -549.50 |
| 5(phy-HPV16/18 triage) | 4.05686 | 3.65979 | 0.17786 | 0.00002 | 8900.17 |  | -0.11254 | 0.00020 | -564.35 |
| 6(phy-HPV7 types triage) | 3.95953 | 3.65993 | 0.08053 | 0.00015 | 523.46 |  | -0.20987 | 0.00033 | -629.73 |
| 7(phy-HPV with genotype triage) | 3.87900 | 3.65977 | / | / | / |  | -0.29040 | 0.00018 | -1618.34 |
| 8(phy-HPV with cytology triage) | 4.16940 | 3.65959 | 0.29040 | -0.00018 | -1618.34 |  | / | / | / |
| Urban areas | | | | | |  |  |  |  |
| 1(self-HPV without triage) | 3.79673 | 3.66537 | -0.47121 | 0.00025 | -1857.18 |  | -0.81843 | 0.00044 | -1856.90 |
| 2(self-HPV16/18triage) | 4.02134 | 3.66517 | -0.24660 | 0.00005 | -4791.69 |  | -0.59382 | 0.00024 | -2489.91 |
| 3(self-HPV7 types triage) | 3.83683 | 3.66532 | -0.43111 | 0.00020 | -2208.16 |  | -0.77833 | 0.00038 | -2036.12 |
| 4(phy-HPV without triage) | 4.28878 | 3.66534 | 0.02084 | 0.00022 | 95.89 |  | -0.32638 | 0.00040 | -807.09 |
| 5(phy-HPV16/18 triage) | 4.45078 | 3.66515 | 0.18284 | 0.00003 | 6964.90 |  | -0.16438 | 0.00021 | -770.75 |
| 6(phy-HPV7 types triage) | 4.30866 | 3.66529 | 0.04072 | 0.00017 | 246.20 |  | -0.30651 | 0.00035 | -869.74 |
| 7(phy-HPV with genotype triage) | 4.26794 | 3.66512 | / | / | / |  | -0.34722 | 0.00019 | -1856.53 |
| 8(phy-HPV with cytology triage) | 4.61516 | 3.66493 | 0.34722 | -0.00019 | -1856.53 |  | / | / | / |
| Rural areas | | | | | |  |  |  |  |
| 1(self-HPV without triage) | 3.27734 | 3.65234 | -0.04495 | 0.00022 | -206.71 |  | -0.25402 | 0.00039 | -657.95 |
| 2(self-HPV16/18triage) | 3.31875 | 3.65216 | -0.00354 | 0.00004 | -95.17 |  | -0.21261 | 0.00021 | -1032.86 |
| 3(self-HPV7 types triage) | 3.25965 | 3.65229 | -0.06264 | 0.00017 | -378.67 |  | -0.27170 | 0.00033 | -813.47 |
| 4(phy-HPV without triage) | 3.48704 | 3.65230 | 0.16475 | 0.00018 | 898.28 |  | -0.04431 | 0.00035 | -125.90 |
| 5(phy-HPV16/18 triage) | 3.49301 | 3.65213 | 0.17072 | 0.00001 | 15504.52 |  | -0.03834 | 0.00018 | -213.50 |
| 6(phy-HPV7 types triage) | 3.45980 | 3.65226 | 0.13751 | 0.00014 | 1001.47 |  | -0.07155 | 0.00031 | -233.93 |
| 7(phy-HPV with genotype triage) | 3.32229 | 3.65212 | / | / | / |  | -0.20906 | 0.00017 | -1240.11 |
| 8(phy-HPV with cytology triage) | 3.53135 | 3.65195 | 0.20906 | -0.00017 | -1240.11 |  | / | / | / |

**Fig. S5a. Tornado diagram analysis for self-HPV without triage versus current physician-HPV with cytology strategy in urban area**

-1856.90

0

-3713.80

-5570.70

-7427.60

-9284.50

Incremental cost-effectiveness ratio($/QALY)

-984.79

-106.79

-7857.95

-5157.95

-1557.95

-657.95

-6057.95

-2457.95

-1557.95

-2457.95

-3357.95

-4257.95

-6957.95

0

**Fig. S5b. Tornado diagram analysis for self-HPV without triage versus current physician-HPV with cytology strategy in rural area**

**References**

1. Xia C, Hu S, Xu X, et al. Projections up to 2100 and a budget optimisation strategy towards cervical cancer elimination in China: a modelling study. *The Lancet Public Health* 2019; 4: e462-e72.

2. Xia C, Xu X, Zhao X, et al. Effectiveness and cost-effectiveness of eliminating cervical cancer through a tailored optimal pathway: a modeling study. *BMC Med* 2021; 19: 62.

3. ICO/IARC Information Centre on HPV and Cancer. Human papillomavirus and related diseases in China: HPV Information Centre. 2018. https://hpvcentre.net/. Accessed 18 October 2022.

4. National Bureau of Statistics of China. China population and employment statistics yearbook 2016. Beijing: China Statistics Press; 2017.

5. National Health and Family Planning Commission. 2016 China health statistics yearbook. Beijing: Peking Union Medical College Press. 2016.

6. He J. 2018 China Cancer Registry Annual Report. Beijing: People’s Medical Publishing House; 2019.

7. National Bureau of Statistics of China. National data. http://data.stats.gov.cn/english/ Accessed 21 October 2022.

8. Dong L, Hu SY, Zhang Q, et al. Risk prediction of cervical cancer and precancers by type-specific human papillomavirus: evidence from a population-based cohort study in China. *Cancer Prev Res (Phila)* 2017;10:745-751.

9. Haeussler K, den Hout AV, Baio G. A dynamic Bayesian Markov model for health economic evaluations of interventions in infectious disease. *BMC Med Res Methodol* 2018; 18: 82.

10. Haeussler KD. A dynamic Bayesian Markov model for health economic evaluations of interventions in infectious disease. London: UCL (University College London); 2017.

11. Liu YJ, Zhang Q, Hu SY, Zhao FH. Effect of vaccination age on cost-effectiveness of human papillomavirus vaccination against cervical cancer in China. *BMC Cancer* 2016; 16: 164.

12. Canfell K, Barnabas R, Patnick J, Beral V. The predicted effect of changes in cervical screening practice in the UK: results from a modelling study. *Br J Cancer* 2004; 91: 530-6.

13. Goldie SJ, Grima D, Kohli M, Wright TC, Weinstein M, Franco E. A comprehensive natural history model of HPV infection and cervical cancer to estimate the clinical impact of a prophylactic HPV-16/18 vaccine. *Int J Cancer* 2003; 106: 896-904.

14. Haeussler K, Marcellusi A, Mennini FS, et al. Cost-Effectiveness Analysis of Universal Human Papillomavirus Vaccination Using a Dynamic Bayesian Methodology: The BEST II Study. *Value Health* 2015; **18**: 956-68.

15. Myers ER, McCrory DC, Nanda K, Bastian L, Matchar DB. Mathematical model for the natural history of human papillomavirus infection and cervical carcinogenesis. *Am J Epidemiol* 2000; 151: 1158-71.

16. Masatoshi Y, Tsuyoshi I, Chisato N, et al. Prognostic factors associated with the clinical outcome of cervical intraepithelial neoplasia: a cohort study in Japan. *Cancer Lett* 2003; 192: 171-9.

17. Sawaya GF, Sanstead E, Alarid-Escudero F, et al. Estimated quality of life and economic outcomes associated with 12 cervical cancer screening strategies: A cost-effectiveness analysis. *JAMA Intern Med* 2019; 179: 867-78.

18. Zhao XL, Xu XQ, Duan XZ, et al. Comparative performance evaluation of different HPV tests and triaging strategies using self-samples and feasibility assessment of thermal ablation in 'colposcopy and treat' approach: A population-based study in rural China. *Int J Cancer* 2020; 147: 1275-85.

19. Rezhake R, Chen F, Hu SY, et al. Triage options to manage high-risk human papillomavirus-positive women: A population-based cross-sectional study from rural China. *Int J Cancer* 2020; 147: 2053-64.

20. J L Belinson, Y L Qiao, R G Pretorius, et al. Shanxi Province cervical cancer screening study II: Self-sampling for high-risk human papillomavirus compared to direct sampling for human papillomavirus and liquid based cervical cytology. *Int J Gynecol Cancer* 2003; 13: 819-26.

21. Zhao XL, Liu ZH, Zhao S, et al. Efficacy of point-of-care thermal ablation among high-risk human papillomavirus positive women in China. *Int J Cancer* 2021; 148: 1419-27.

22. Santesso N, Mustafa RA, Wiercioch W, et al. Systematic reviews and meta-analyses of benefits and harms of cryotherapy, LEEP, and cold knife conization to treat cervical intraepithelial neoplasia. *Int J Gynaecol Obstet* 2016; 132: 266-71.

23. Yeh PT, Kennedy CE, de Vuyst H, Narasimhan M. Self-sampling for human papillomavirus (HPV) testing: a systematic review and meta-analysis. *BMJ Glob Health* 2019; 4: e001351.

24. Goldie SJ, Gaffikin L, Goldhaber-Fiebert JD, et al. Cost-effectiveness of cervical-cancer screening in five developing countries. *N Engl J Med* 2005; 353: 2158-68.

25. National Bureau of Statistics. China's 6th National Population Census. <http://www.stats.gov.cn/tjsj/pcsj/rkpc/6rp/indexch.htm>. Accessed 12 October 2022.

26. China Hainan Government Procurement. Hainan Women's and Children's Medical Center - HPV testing reagents and related services for women's common diseases and "two cancers" in 2019 -bid winning announcement. <https://www.ccgp-hainan.gov.cn/cgw/cgw_show_zbgg.jsp?id=21831>. Accessed 12 October 2022.

27. Zhejiang Government Procurement Center. Announcement of bid winning (transaction) results of "two cancer" examination project of provincial Health Commission, HPV and TCT test reagent project. <http://zfcg.czt.zj.gov.cn/innerUsed_noticeDetails/index.html?noticeId=6604290>. Accessed 15 October 2022.

28. Jiangsu Public Resource Trading Platform. TCT and HPV test service project of "two cancers" program in Tongzhou District, Nantong City. <http://jsggzy.jszwfw.gov.cn/jyxx/003004/003004002/20200421/f67ce97c-e4f6-44b3-977e-4bc17d5930df.html>. Accessed 15 October 2022.

29. Peng JR, Tao SY, Wen Y, Yang X, Ma JQ, Zhao F. Cost-effectiveness analysis of cervical cancer screening strategies in urban China. *Chinese Journal of Oncology* 2019; 41:154-60.

30. Zhao FH, Adam KL, Hu SY, et al. Prevalence of human papillomavirus and cervical intraepithelial neoplasia in China: a pooled analysis of 17 population-based studies. *Int J Cancer* 2012; 131: 2929-38.

31. National Bureau of Statistics of China. Residents' income and consumption expenditure in 2019. 2020. <http://www.stats.gov.cn/tjsj/zxfb/202001/t20200117_1723396.html>. Accessed 15 October 2022.

32. Tao SY, Peng JR, Wang Y, et al. Study on direct economic burden and influencing factors in patients with cervical cancer and precancerous lesions. *Chi J Prev Med* 2018; 52: 1281-6.

33. Ma L, Wang Y, Gao X, et al. Economic evaluation of cervical cancer screening strategies in urban China. *Chin J Cancer Res* 2019; 31: 974-83.

34. Wen TM, Zhao S, Zhao XL, et al. Investigation on quality of life of women with different grades of cervical lesions and treatments. *Chin J Clin Obstet Gynecol* 2023;24:144-148..

35. Luo N, Liu G, Li M, Guan H, Jin X, Rand-Hendriksen K. Estimating an EQ-5D-5L Value Set for China. *Value Health* 2017; 20: 662-9.
